# Construction and Validation of a Novel Glycometabolism-Related Gene Signature Predicting Survival in Patients With Ovarian Cancer

**DOI:** 10.3389/fgene.2020.585259

**Published:** 2020-11-12

**Authors:** Lixiao Liu, Luya Cai, Chuan Liu, Shanshan Yu, Bingxin Li, Luyao Pan, Jinduo Zhao, Ye Zhao, Wenfeng Li, Xiaojian Yan

**Affiliations:** ^1^Department of Obstetrics and Gynecology, The First Affiliated Hospital of Wenzhou Medical University, Wenzhou, China; ^2^Department of Medical Oncology, The First Hospital of China Medical University, Shenyang, China; ^3^Department of Chemoradiation Oncology, The First Affiliated Hospital of Wenzhou Medical University, Wenzhou, China

**Keywords:** ovarian cancer, glycometabolism, prognosis, gene signature, PCR

## Abstract

Among all fatal gynecological malignant tumors, ovarian cancer has the highest mortality rate. The purpose of this study was to develop a stable and personalized glycometabolism-related prognostic signature to predict the overall survival of ovarian cancer patients. The gene expression profiles and clinical information of ovarian cancer patients were derived from four public GEO datasets, which were divided into training and testing cohorts. Glycometabolism-related genes significantly associated with prognosis were selected. A risk score model was established and validated to evaluate its predictive value. We found 5 genes significantly related to prognosis and established a five-mRNA signature. The five-mRNA signature significantly divided patients into a low-risk group and a high-risk group in the training set and validation set. Survival analysis showed that high risk scores obtained by the model were significantly correlated with adverse survival outcomes and could be regarded as an independent predictor for patients with ovarian cancer. In addition, the five-mRNA signature can predict the overall survival of ovarian cancer patients in different subgroups. In summary, we successfully constructed a model that can predict the prognosis of patients with ovarian cancer, which provides new insights into postoperative treatment strategies, promotes individualized therapy, and provides potential new targets for immunotherapy.

## Introduction

Ovarian cancer (OC) is one of the three types of gynecological malignant tumors. According to global cancer statistics, there were approximately 290,000 new cases of cervical cancer worldwide in 2018, resulting in 180,000 deaths (Bray et al., 2018). Due to the lack of specific clinical symptoms and screening methods as well as its insidious onset, nearly 70% of OC patients are diagnosed at an advanced stage (Partridge et al., 2009; Chudecka-Głaz, 2015), which leads to approximately 70% of patients with local recurrence or distant metastasis after standard treatment, and the clinical treatment effect of cancer is not satisfactory due to drug resistance (Jayson et al., 2014; Giornelli, 2016). Despite great progress in clinical treatment, including surgery, targeted therapy, chemotherapy and radiotherapy, the 5 years overall survival rate of OC is only 30% (Bray et al., 2018). In clinical practice, histopathology and CA-125 are often used to predict the prognosis of patients with OC (Mittermeyer et al., 2013; Yuan et al., 2017; Zwakman et al., 2017; Hua et al., 2019). However, these methods have their own limitations. In clinical practice, doctors often use the same treatment for patients with the same clinical stage and pathological type; however, the clinical outcomes are usually different because of the heterogeneity of tumors.

With the development of high-throughput sequencing, several patient genome databases have been established, which have enabled us to gain a more systematic and comprehensive understanding of patients’ genome changes. By mining databases, we identified a number of biomarkers associated with clinical outcomes (Buttarelli et al., 2020; Nash and Menon, 2020; Zhang et al., 2020; Zhou et al., 2020). However, a single gene cannot accurately predict the outcomes of OC. In contrast, the evaluation of multiple gene combinations may be able to better predict the prognosis of patients and guide clinicians in selecting more appropriate treatment options and intervening with patients earlier to prolong their survival time.

Current studies have shown that malignant tumors are related not only to genetic information but also to their own energy metabolism (Hanahan and Weinberg, 2011). The most striking effect is the Warburg effect (Warburg, 1956a,b). Even under the condition of normal oxygen content, tumor cells still use glycolysis as a source of energy metabolism, which is characterized by high glucose uptake and active glycolysis, which converts glucose into lactic acid to produce ATP. Therefore, the inhibition of glycolysis can inhibit the proliferation of tumor cells and kill tumor cells, and the rate-limiting enzymes of glycolysis and hypoxia-inducible factors are expected to become new targets for the treatment of tumors. Previous studies have shown that glycolysis is related to the activation of oncogenes (such as RAS and MYC) and mutations of tumor suppressor genes (such as TP53) (DeBerardinis et al., 2008; Dejana et al., 2009). Specifically, the overexpression of MYC can lead to an imbalance in the translation mechanism. RAS and c-MYC affect translation and metabolism by stimulating glycolysis and disrupting protein synthesis (Biffo et al., 2018). P53 is a tumor suppressor gene in the human body that plays a core role in metabolic pathways. TP53 mutations are reported in at least 80% of advanced serous OC cases, and the poor prognosis associated with TP53 mutations is thought to be due to the resistance of cancer cells to chemotherapy-induced apoptosis (Duffy et al., 2017). It has been reported that the increased glucose metabolism caused by the upregulation of HK II can inhibit the activity of p53 (Han et al., 2019), which suggests that the high level of glycolysis in tumor cells may contribute to the downregulation of the antitumor effect of p53. Other studies have shown that SIK2 can upregulate HIF-1 phosphorylation by activating the PI3K/AKT signaling pathway and promote mitochondrial division by phosphorylating Drp1 at the Ser616 site, thereby promoting glycolysis and inhibiting mitochondrial oxidative phosphorylation (Gao et al., 2020). Elucidating the relationship between glucose metabolism and tumors is critical for us to understand the mechanisms of tumorigenesis and development, and it is of great clinical significance to construct a model that can accurately predict OC prognosis based on glycometabolism-related genes.

In this study, we integrated the gene expression profiles of 380 patients with OC from the GEO database, screened glycometabolism-related genes closely related to patient prognosis, and constructed a risk score model based on 5 glycometabolism-related genes to predict the prognosis of OC patients individually. With external validation of the independent dataset, we demonstrated the model’s accuracy and reliability. In addition, we conducted a comprehensive analysis of this model with clinical characteristics to improve the accuracy of overall survival prediction.

## Materials and Methods

### Acquirement of Public Gene Expression Profiles and Glycometabolism-Related Genes

Gene expression profiles were downloaded from the GEO database, and the nucleic acid sequences of 380 OC patients were downloaded from the GSE140082 dataset as the training set. Clinical data such as age, stage, treatment, and OS were also obtained from the GEO database. A total of 380 patients with OC were included in the analysis. Table 1 shows the clinical information of the included patients. Moreover, the gene expression profiles and clinical information of patients were obtained from the GSE17260, GSE 26712 and GSE32062 datasets as the testing group, respectively. In addition, we searched for “glycolysis” in GSEA^1^ to select the set of genes most closely related to glucose metabolism. Six glycometabolism-related gene sets were obtained from GSEA to identify glycometabolism-related genes.

**TABLE 1:** Clinicopathological parameters of the ovarian cancer patients in this study.

**Clinical characteristic**	***N***	**%**
**Age(years)**		
≤65	275	72.3
>65	105	27.6
**FIGO stage**		
I–II	51	13.4
III–IV	329	86.6
**Treatment**		
Standard	181	47.6
Bevacizumab	199	52.4
**Grade**		
High	281	73.9
Low	74	19.5
**Molecular subgroup**		
Immunoreactive	124	32.6
Proliferative	97	25.5
Differentiated	86	22.6
Mesenchymal	73	19.2
**Outcome of surgery**		
Optimal	290	76.3
Sub-optimal	88	23.1

### Arrangement of the Gene Expression Database

The datum have been standardized by the original data providers. According to the annotation file, the gene probe of each gene expression profile was converted to the corresponding gene name. In this study, only patients with complete overall survival (OS), staging and other clinical information were selected.

### Pathway Enrichment Analysis to Identify Molecular Functions

Furthermore, to better comprehend the gene functions of all of the glycometabolism-related genes that we obtained from GESA, pathway enrichment analysis of these genes was carried out. We used the clusterProfiler package to analyze the signaling pathways of all the related genes through the Kyoto Encyclopedia of Genes and Genomes (KEGG) database and also analyzed their biological processes (BPs), molecular functions (MFs) and cellular components (CCs) through the Gene Ontology (GO) database in the R program. The aim was to determine whether the screened genes were indeed associated with glucose metabolism.

### Construction of a Glycometabolism-Related Signature for OC

We established a glycometabolism prognostic model using the GEO database and glycometabolism-related gene set. The expression profiles of 20,790 mRNAs were taken as the original data. First, we converted the gene expression profiles of GSE140082 from the gene probe to the corresponding gene name. These genes were intersected with the glycometabolism-related genes that were obtained from six glycometabolism-related gene sets, and univariate Cox analysis was performed on the candidate genes (*P* < 0.01). The purpose was to identify genes that were clearly associated with overall survival. Simultaneously, to screen genes with higher correlations and to prevent overfitting of the model, we used the glmnet package for the LASSO algorithm to reduce the number of candidate genes to 10. Then, stepwise multivariate Cox regression analysis was carried out to determine the most valuable genes related to glycometabolism. We eventually obtained five glycometabolism-related genes associated with prognosis to construct a predictive risk score model. Risk score = expression of B3GAT3 ^∗^ (−0.136052404) + expression of COL5A1 ^∗^ 0.31010011 + expression of FAM162A ^∗^ 0.424815423 + expression of IDUA ^∗^ (−0.091168453) + expression of PPP2R1A ^∗^ (−0.210503843). The patients were divided into a high-risk group and a low-risk group according to the median risk score. Finally, the survival package was used for survival analysis, and Kaplan-Meier (K-M) curves and ROC curves were drawn by the survival ROC package to evaluate the difference in survival outcomes between the high-risk group and the low-risk group. Moreover, univariate and multivariate analyses were used to assess the impact of risk scores and other clinical features on overall survival.

### External Validation of the Five-mRNA Signature

To verify the predictive prognostic value of the glycometabolism-related gene risk model, we used the GSE17260, GSE 26712, and GSE32062 datasets as the validation cohorts. The same formula was used to calculate the risk score, and the patients were divided into high-risk and low-risk groups based on the median value. A K-M curve and ROC curve were drawn to analyze the data.

### Cell Culture

The human ovarian cancer SKOV-3 and A2780 cells and HOSEpiC human ovarian epithelial cells were maintained in adherent culture conditions. A2780 and SKOV-3 cells were cultured in RPMI 1640 medium (Gibco, Grand Island, NY, United States), and HOSEpiC cells were cultured in DMEM (Gibco) supplemented with 10% fetal bovine serum (FBS) and 1% antibiotic-antimycotic solution. All cell lines were grown in a humidified incubator at 37°C (5% CO_2_).

### Detection of Gene Expression Levels of Five Genes in OC

Total RNA was extracted from the cells using TRIzol reagent (Thermo Fisher Scientific, Waltham, MA, United States). Single-stranded cDNA was synthesized from 1 mg of total RNA using the PrimeScript RT Reagent Kit with gDNA Eraser (Takara Biotechnology Co., Ltd., Dalian, China).

Reverse transcription quantitative PCR was used to detect the mRNA expression of the hub genes by a 7500 PCR system (Thermo Fisher Scientific). The primers used are shown in Table 2. The following cycling conditions were used: 95°C for 5 min, followed by 40 cycles of 95°C for 20 s and 60°C for 30 s. qPCR assays were conducted in triplicate in a 10 mL reaction volume for each sample. The relative expression of B3GAT3, COL5A1, FAM162A, IDUA, and PPP2R1A mRNA was calculated by the 2-Ct method.

**TABLE 2:** The primers for the five genes.

**Primer name**	**Forward sequence**	**Reverse sequence**
B3GAT3	GCCCTTGCTGTTAGATAAGCCC	GCTTCTCTGTCCGAGTATGCCA
COL5A1	GGAGATGATGGTCCCAAAGGCA	CCATCATCTCCTTTGTCACCAGG
FAM162A	ATTGCCCTGACGGTGGTAGGAT	CTGCTTCCTCTTTCAGACGAGC
IDUA	CAGCAGGTGTTTGAGTGGAAGG	GGAGACGTTGTCAAAGTCGTGG
PPP2R1A	ACCGCATGACTACGCTCTTCTG	TTGAAGCGGACATTGGCAACCG

### Statistical Analysis

All analyses were performed using Rversion 3.6.2. All statistical tests were two-sided. Unless otherwise specified, *P* < 0.05 was considered significant.

## Results

### Screening of Glycometabolism Genes

The gene expression profiles and clinical information of 380 patients were obtained from the GSE140082 dataset. We first downloaded six sets of glycometabolism-related genes from GSEA. Namely, six different gene sets (BIOCARTA_ GLYCOLYSIS_PATHWAY, HALLMARK_GLYCOLYSIS, KEGG_ GLYCOLYSIS_GLUCONEOGENESIS,MODULE_306, REACTOME_GLYCOLYSIS, and REACTOME_REGULATION_ OF_GLYCOLYSIS_BY_FRUCTOSE_2_6_BISPHOSPHATE_ METABOLISM) were used. Then, the transcriptome matrix of 380 patients was intersected to obtain the expression of 289 glycometabolism-related genes.

### Identification of Glycometabolism-Related Genes

**FIGURE 1:**
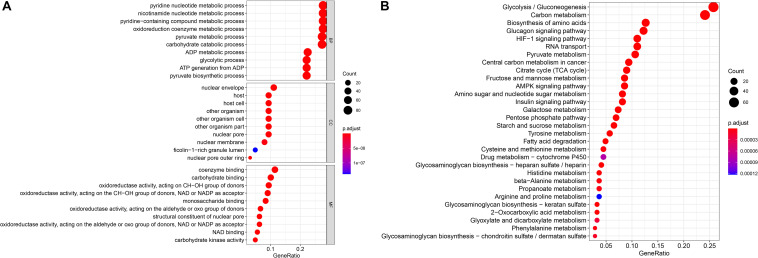
Gene functional enrichment of the 289 specific glycometabolism-related genes. **(A)** Gene Ontology analysis; BP,CC and MF represent biological process, cellular component and molecular function, respectively. **(B)** The most significant Kyoto Encyclopedia of Genes and Genomes (KEGG) pathways.

To ensure that the genes present in the screening were indeed associated with glycometabolism, we used GO and KEGG enrichment analyses to analyze the 289 glycometabolism-related genes. GO analysis revealed that the primary functions enriched in the biological process (BP) category were pyridine nucleotide metabolic process, nicotinamide nucleotide metabolic process, pyridine–containing compound metabolic process, oxidoreduction coenzyme metabolic process, pyruvate metabolic process, and carbohydrate catabolic process. For cellular components (CCs), the nuclear envelope was the mainly enriched GO term. For molecular functions (MFs), coenzyme binding, carbohydrate binding and oxidoreductase activity, and acting on the CH–OH group of donors were the most enriched (Figure 1A). For the KEGG pathways, glycolysis/gluconeogenesis and carbon metabolism were most often enriched by the glycometabolism-related genes (Figure 1B). These results indicated that the screened candidate genes were indeed related to glycometabolism.

### Identification of Glycometabolism Genes Related to the Survival of OC Patients

**FIGURE 2:**
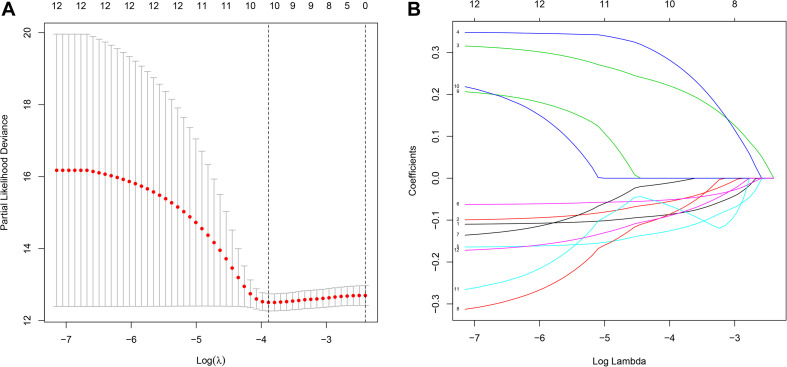
Construction of the prognostic prediction model for patients with ovarian cancer by LASSO. **(A)** The changing trajectory of each independent variable. The horizontal axis represents the log value of the independent variable lambda, and the vertical axis represents the coefficient of the independent variable. **(B)** Confidence intervals for each lambda. The horizontal axis represents the log value of the independent variable lambda, and the vertical axis represents the error of cross-verification.

**FIGURE 3:**
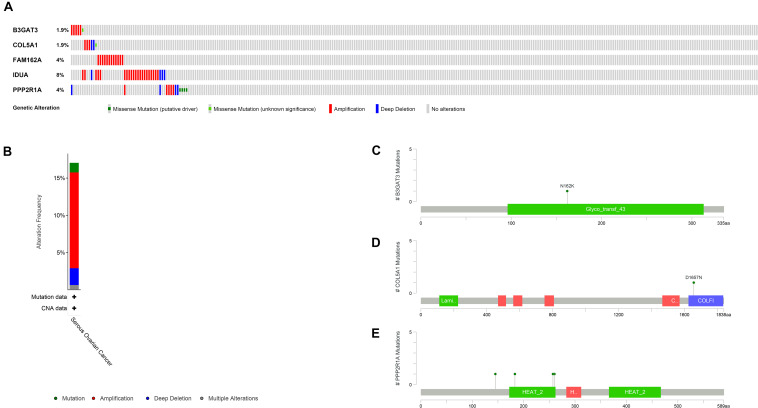
Identification of a prognostic risk signature associated with glycometabolism. **(A)** Selected gene alterations in 311 clinical samples. **(B)** Specific alteration frequencies of the selected gene sequences of the clinical samples. **(C)** Mutations in B3GAT3. **(D)** Mutations in COL5A1. **(E)** Mutations in PPP2R1A.

To identify novel genetic biomarkers related to the prognosis of patients with OC, we first performed univariate Cox analysis of 289 genes enriched by glycometabolism, and a total of 12 genes were significantly associated with OS (*P* < 0.01). Moreover, to screen genes with higher correlations and to prevent overfitting of the model, we used the LASSO algorithm to reduce the number of candidate genes to 10 (Figure 2), and these genes were included in a stepwise multivariate Cox regression analysis. Five genes independently associated with OS were finally obtained by the multivariate Cox analysis (B3GAT3, COL5A1, FAM162A, IDUA, and PPP2R1A). Then, we analyzed the changes in the five candidate genes in 311 clinical OC samples from the cBioPortal database. The results showed that the genetic sequences of 62 patients (17.04%) had changed (Figure 3A). Specifically, 1.29% had mutations, 12.86% had amplifications, 2.25% had deep deletions and 0.64% had multiple alterations (Figure 3B). Specific alterations in the genes that were selected were also distinct in OC. In the B3GAT3 gene, there was a mutation in the Glycosyl transferase family 43 (Glyco_transf_43) structural domain, resulting in a change in N162K (Figure 3C). In the COL5A1 gene, there was a mutation in the fibrillar collagen C-terminal domain (COLFI) structural domain, resulting in a change in D1657N (Figure 3D). At the same time, there were three mutations in the HEAT repeats (HEAT_2) structural domain in the PPP2R1A gene, namely, R183W, R260C and W257C, and T145P appeared outside the structural domain (Figure 3E). We hypothesized that the changes in proteins in the domain might affect the function of the structural domain.

### Construction of a Five-mRNA Signature to Predict Patient Prognosis

**FIGURE 4:**
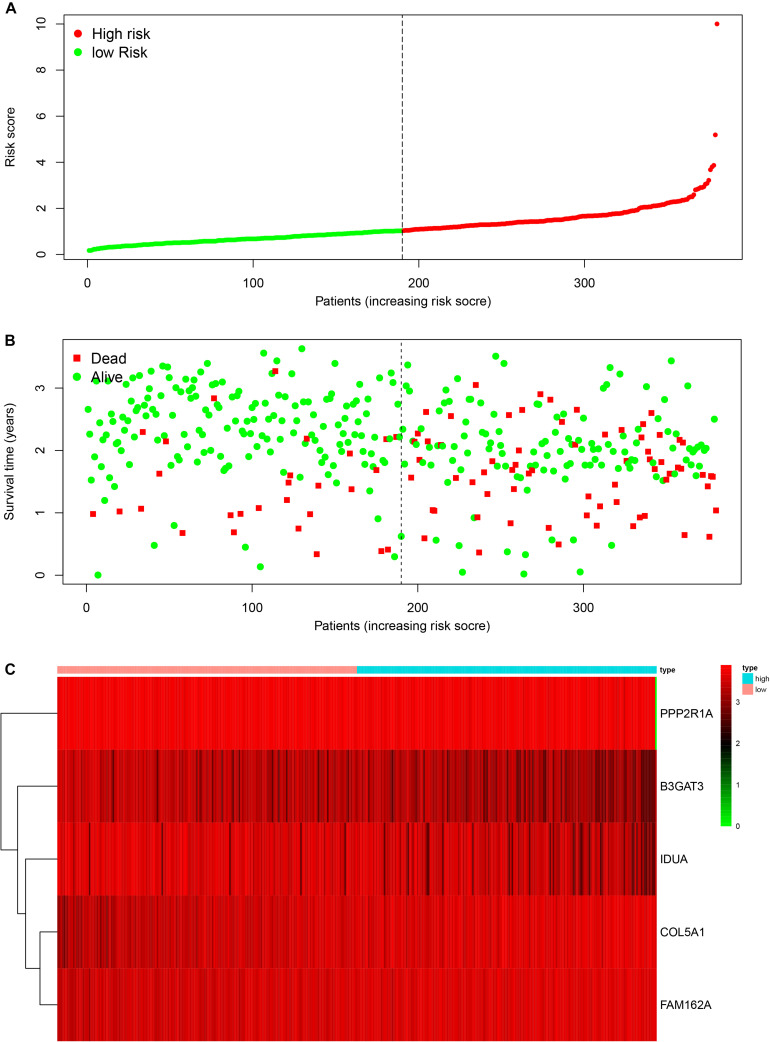
The relationship between the risk score and patient outcomes. **(A)** The risk value of each ovarian cancer patient in the GSE140082 dataset. **(B)** The survival status and survival time of patients in different groups. **(C)** Heatmap of the expression profiles of 5 glycometabolism-related genes in OC patients.

**FIGURE 5:**
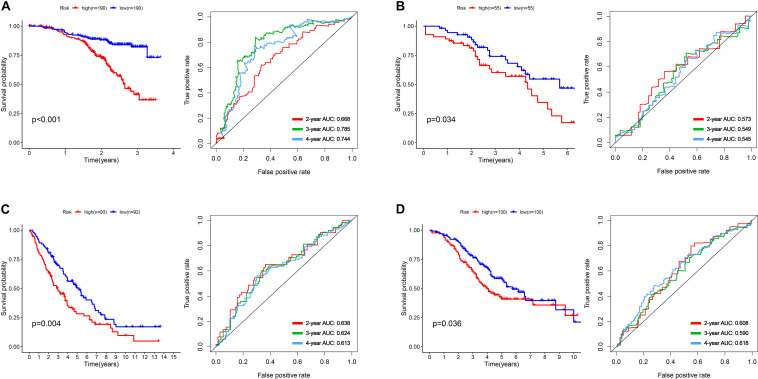
The development and validation of the five-mRNA signature. **(A)** Kaplan-Meier analysis and ROC curves of overall survival in the high- and low-risk groups of OC patients in the GSE140082 dataset. **(B)** Kaplan-Meier analysis and ROC curves of overall survival in the high- and low-risk groups of OC patients in the GSE17260 dataset. **(C)** Kaplan-Meier analysis and ROC curves of overall survival in the high- and low-risk groups of OC patients in the GSE 26712 dataset. **(D)** Kaplan-Meier analysis and ROC curves of overall survival in the high- and low-risk groups of OC patients in the GSE32062 dataset.

To construct a prognostic model, we used the five glycometabolism-related genes to evaluate the survival risk of each patient. Risk score = expression of B3GAT3 ^∗^ (−0.136052404) + expression of COL5A1 ^∗^ 0.31010011 + expression of FAM162A ^∗^ 0.424815423 + expression of IDUA ^∗^ (−0.091168453) + expression of PPP2R1A ^∗^ (−0.210503843). Then, we divided the patients according to their risk score (Figure 4A) using the median risk score value as the threshold: the 380 patients were divided into a high-risk group (*N* = 190) and a low-risk group (*N* = 190). The survival status of the patients and the expression of the five glycometabolism-related genes are shown in Figures 4B,C. As shown in the figure, with the increase in risk score, the mortality rate of patients in the high-risk group was significantly higher than that in the low-risk group, and the survival time was generally lower than that in the low-risk group. Kaplan-Meier (K-M) analysis revealed significant differences in prognosis between the high-risk and low-risk groups (Figure 5A, *P* < 0.001). The survival rate in the low-risk group was significantly higher than that in the high-risk group. We drew a ROC curve to indicate the accuracy of the model, and the area under the curve (AUC) values of the model for 2, 3, and 4 years were 0.668, 0.785, and 0.744, respectively (Figure 5B), which demonstrated that the model had good sensitivity and specificity for survival prediction.

### External Validation of the Five-mRNA Signature in Other GEO Datasets

We used the gene expression profiles and clinical information of patients from the GSE17260, GSE 26712, and GSE32062 datasets as the testing sets. According to the median risk score, the patients were divided into a high-risk group and a low-risk group. The results were consistent with those of the training group (Figures 5B–D). The survival rate of patients in the high-risk group in the validation group was significantly lower than that in the low-risk group (*P* = 0.034, 0.004, 0.036). The AUC value of the model for 2, 3, and 4 years were showed in the pictures, which proved the robust effectiveness of the five-mRNA signature in predicting OS.

### The Five-mRNA Signature Is an Independent Prognostic Factor for OC

**FIGURE 6:**
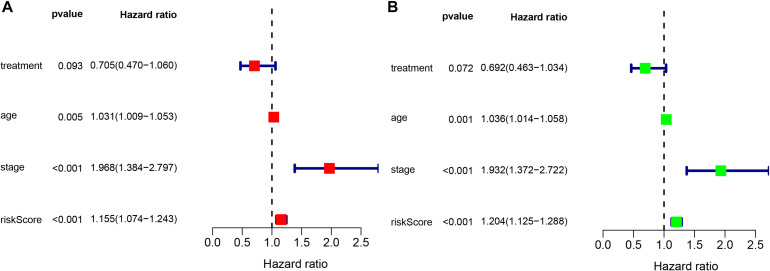
Univariate and multivariate Cox analyses evaluating the independent predictive ability of our signature for OS. **(A)** Univariate Cox analyses and **(B)** multivariate Cox analyses.

**FIGURE 7:**
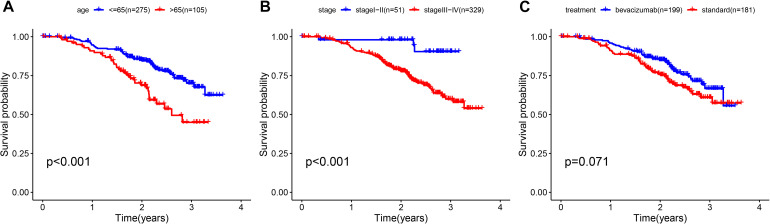
Clinical features, including age, stage, and treatment, predict patient survival. **(A)** Age, **(B)** stage, and **(C)** treatment.

**FIGURE 8:**
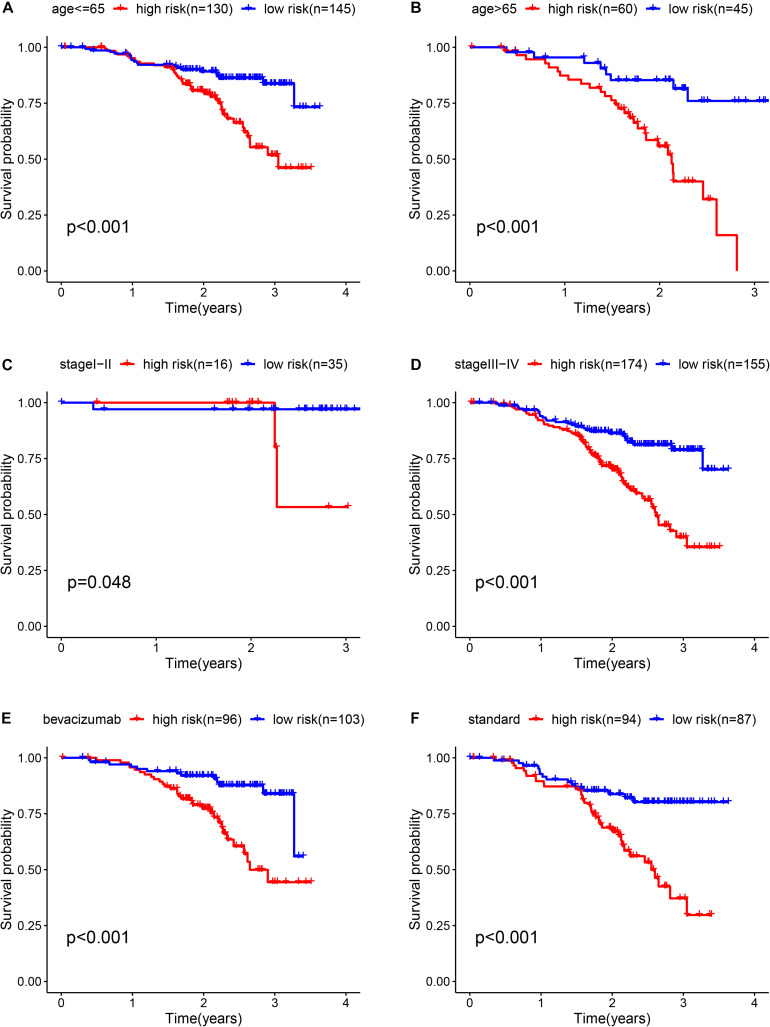
Kaplan-Meier curves for the prognostic value of the risk score signature for the patients grouped according to each clinical feature. **(A)** Age ≤ 65, **(B)** Age > 65, **(C)** stage I-II, **(D)** stage III-IV, **(E)** bevacizumab, and **(F)** Standard.

To determine whether the prognostic ability of the five-mRNA signature is independent of various clinical features, including treatment mode, age and stage, we further used univariate and multivariate Cox analyses. Univariate Cox analysis showed that age, stage and risk score were correlated with OS in OC patients. After adjusting for other clinical parameters in the dataset, the five-mRNA signature remained a significant independent prognostic indicator (*P*-value < 0.001, HR = 1.155, 95% CI = 1.074–1.243, Figure 6). In addition, we found that age and stage were significantly different in univariate and multivariate Cox analyses and were also independent prognostic factors. However, compared with the standard treatment (paclitaxel + carboplatin), whether bevacizumab is added or not had no significant prognostic effect and was not found to be an independent prognostic factor. Then, we divided the patients into groups according to their age, stage and treatment mode and used the K-M survival curve for further analysis, and similar results were obtained. Age over 65 and stage III–IV were associated with poor prognosis in patients (Figure 7), which further validated the dependability of the model. After further data mining and stratified analysis, we found that the survival curve was not affected by age, stage or treatment (Figure 8), confirming that the five-mRNA signature can be used to predict the outcomes of patients with OC.

### Exploration of the Specific Roles of the Candidate Genes in Tumorigenesis by RT-PCR

**FIGURE 9:**
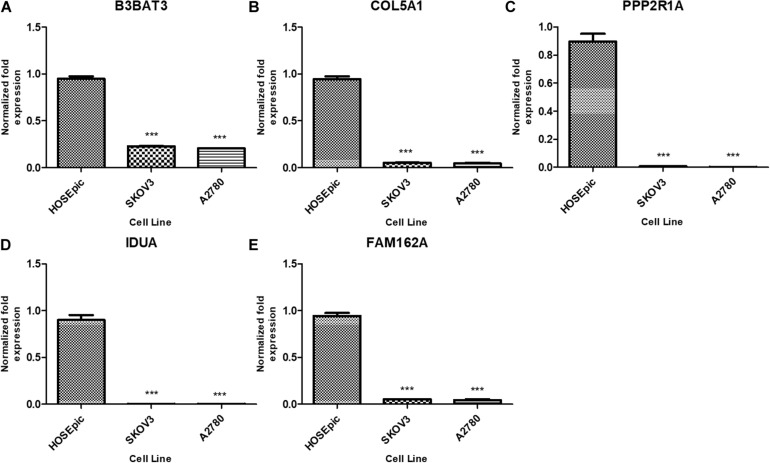
Expression of five mRNAs in ovarian cancer cells and human ovarian epithelial cells. **(A)** B3GAT3, **(B)** COL5A1, (**C)** PPP2R1A, **(D)** IDUA, **(E)** FAM162A. Compared to normal groups, ***P < 0.001.

We further analyzed the differential expression of B3GAT3, COL5A1, FAM162A, IDUA, and PPP2R1A in ovarian cancer SKOV-3 and A2780 cells and HOSEpiC human ovarian epithelial cells. The RT-qPCR results showed that compared with human ovarian epithelial cells, the expression of B3GAT3, COL5A1, FAM162A, IDUA, and PPP2R1A in ovarian cancer cells was significantly downregulated (Figure 9).

## Discussion

In recent years, features such as age, pathological stage, lymph node metastasis and distant metastasis have been commonly used in clinical work to predict the prognosis of patients with OC (Mittermeyer et al., 2013; Yuan et al., 2017; Zwakman et al., 2017; Hua et al., 2019), but the accuracy of these factors is insufficient. With the development of high-throughput sequencing technology, an increasing number of mRNAs have been discovered as biomarkers that can be used to evaluate and predict the prognosis of OC (Buttarelli et al., 2020; Nash and Menon, 2020; Zhang et al., 2020; Zhou et al., 2020). For instance, some scholars found that the high expression of TET3 was related to the progression of OC and the poor prognosis of patients (Cao et al., 2019). Other scholars found that the upregulated expression of the CDCA gene family in OC may play important roles in the occurrence and development of OC (Chen et al., 2020). However, these genes cannot be used to predict the survival rate of patients because various factors can affect the same gene in a variety of ways, resulting in a deviation in the predictive effect. Therefore, an increasing number of researchers began to evaluate the prognosis of patients through models formed by a combination of multiple gene markers (Subramanian and Simon, 2010). Currently, many researchers are focusing on the roles of metabolic factors in the development and progression of tumors (Wang and Li, 2020). Glycolysis is a significant part of glycometabolism, but there is no prognostic model based on glycometabolism-related genes for evaluating the prognosis of patients with OC. Therefore, the construction of a predictive model that can accurately predict the prognosis of patients with OC has vital clinical significance.

In this study, we performed bioinformatics analysis with data from the GEO database and obtained 289 glycometabolism-related genes in OC. After univariate and multivariate Cox regression analyses, we identified five significantly independent prognostic genes, including B3GAT3, COL5A1, FAM162A, IDUA, and PPP2R1A. Based on these results, we developed a five-mRNA signature to predict the prognosis of OC. In addition, we validated this signature in the GSE17260 dataset and obtained consistent results, which indicates that the five-mRNA signature can be used as a prognostic indicator in OC. In addition, we analyzed and verified the expression of the five genes in tumor and normal tissues, with the purpose of further exploring the specific roles of the candidate genes in the process of tumor development.

Among the five genes, the protein encoded by beta-1,3-glucuronyltransferase3 (B3GAT3) is a member of the glucuronyltransferase gene family and plays an important role in the biosynthesis of proteoglycan (Barré et al., 2006). Studies have shown that compared with normal tissues, B3GAT3 is upregulated in human hepatocellular carcinoma, and the knockout of the B3GAT3 gene can inhibit cell proliferation, migration and invasion and reverse the process of epithelial mesenchymal transformation (Zhang et al., 2019). Furthermore, it was found that the high expression of B3GAT3 is disadvantageous to the prognosis of human liver cancer and has independent prognostic value in patients with different pathological features of liver cancer (Zhang et al., 2019). However, according to our results, B3GAT3 is expressed at low levels in OC cell lines, and the Coef value of B3GAT3 is −0.136; thus, it is a protective factor that may be related to cancer species and is worthy of further study, This phenomenon may be related to the type of tumor. Collagen type V α1 (COL5A1) encodes the alpha 1 chain of collagen V and is involved in extracellular matrix formation (Zhang et al., 2018). Previous studies have shown that COL5A1 may affect the development of a variety of cancers, such as breast cancer, gastric cancer, lung adenocarcinoma, oral squamous cell carcinoma and ovarian cancer (Chai et al., 2016; Zhao et al., 2016; Li et al., 2017; Sun et al., 2017; Liu et al., 2018); moreover, the expression level of COL5A1 was an independent prognostic factor (Boguslawska et al., 2016). Recent studies have shown that COL5A1 is of high value in the prediction of breast cancer (Ritelli et al., 2013; Riches et al., 2014). At the protein level, the HPA database confirmed that the expression of COL5A1 in breast cancer was higher than that in normal tissues, which was consistent with the results of immunohistochemistry; moreover, the COL5A1 mutation was negatively correlated with the prognosis of breast cancer patients (Wu et al., 2019). In view of the findings of the above studies, we conclude that COL5A1 plays an important role in cancer, especially in OC, and the upregulation of COL5A1 expression will increase cisplatin resistance in cancer cells (Yu et al., 2014). According to the PCR results, we found that COL5A1 in OC cells was generally downregulated, what’s more, SKOV-3 and A2780 were not resistant cells, indicating that cisplatin resistance may be more related to the tumor microenvironment of the human body, which is worth further study. COL5A1 could be a potential therapeutic target, maybe we can change the prognosis of patients by affecting the tumor microenvironment which has important guiding significance for clinical work. FAM162a is known as an HIF-1 alpha-responsive pro-apoptotic molecule (Mazzio and Soliman, 2012). When FAM162A was overexpressed, the hypoxia signal was sent directly to the mitochondria, which led to cell death (Kim et al., 2006). In the field of neurological research, the overexpression of FAM162A causes brain injury by activating caspase-3 to transfer apoptosis-inducing factor into the nucleus where programmed cell death and chromatin condensation occur, leading to ischemia and hypoxia (Qu et al., 2009). However, we did not find that this gene was studied in a cancer-related field, and according to our results, when FAM162A was expressed at high levels, it was detrimental to the prognosis of patients. Iduronidase (IDUA) is an enzyme in lysosomes that participates in the hydrolysis process. Its deficiency will lead to a severe genetic disease type called mucopolysaccharidosis I (MPS-I) (Osborn et al., 2008, 2011). At present, the role of IDUA in cancer is not clear, and research on its relationship with cancer is very limited. One study found that the expression level of IDUA was significantly downregulated in breast cancer patients with visceral metastasis compared with those without visceral metastasis (Savci-Heijink et al., 2019). Similar to our findings, IDUA expression levels were higher in HOSEpiC human ovarian epithelial cells as a protective factor, indicating a better prognosis. Therefore, combined with the coef value obtained from the model, we can infer that IDUA is a protective factor. The in-depth study of IDUA is of great significance in the prevention of cancer metastasis. Protein phosphatase 2 scaffold subunit Aalpha (PPP2R1A) is a constant regulatory subunit that encodes protein phosphatase 2 (PP2), which is related to the negative regulation of cell growth and division (Shi, 2009). Studies have found that PPP2R1A has a high-frequency mutation in serous endometrial carcinoma, indicating a poor prognosis (Nagendra et al., 2012), we found that there were more PPP2R1A mutations in ovarian cancer than in other genes. Some studies found that the overexpression or mutation of PPP2R1A in OC and endometrial carcinoma can promote the growth and migration of tumor cells (Jeong et al., 2016). PPP2R1A may be an effective target for personalized therapy. Interestingly, in this study, according to the Coef value of PPP2R1A, we suggest that PPP2R1A is a protective factor. Therefore, we can understand why it is highly expressed in normal ovarian epithelial cells. We speculate that the mutation of the gene is closely related to the tumor microenvironment or that the special metabolic pattern of tumor tissue in the human body affects the mutation of the gene to some extent, thus promoting the growth and migration of tumor cells. We used these five genes to construct a five-mRNA signature to predict the prognosis of patients with OC. In the cell experiments, all genes were expressed in tumor cells at a low level, which contradicted the risk score model. We believe that this conflicting finding is due to the human tumor microenvironment and the special metabolic pattern of tumors in the human body. In our opinion, protective genes and damaging genes together with the tumor microenvironment affect the occurrence and development of tumors. This topic is worthy of further study. Although the five-mRNA signature can provide an effective model for predicting the prognosis of OC, the current research also has some limitations. First, the risk score model is based on the GEO database rather than the TCGA database, and some genes may be left out. Second, since there were no data from normal OC samples in the GSE140082 detection platform GPL14951, we did not look for differentially expressed genes in the course of the study but carried out PCR experiments to show the differential expression of the five genes in tumor and normal tissues. Third, the prediction function of this model in early OC needs to be improved. Currently, we are actively collecting clinical specimens and data for future research.

In summary, we constructed and validated a five-mRNA signature associated with glycometabolism that can predict the prognosis of patients with OC. According to the risk score, the prognosis of the high-risk population is significantly worse. The findings of this study provide a more comprehensive understanding of glycometabolism-related genes that affect the prognosis of patients with OC, and these genes can more accurately predict the prognosis of patients with OC. These results may provide a new perspective for the study and individualized treatment of OC, have important clinical significance, will help future clinical workers to determine new treatment methods for OC and will provide more gene targets for the treatment of OC patients.

## Data Availability Statement

Publicly available datasets were used in this study. These can be found here: GEO database, accession GSE140082, GSE17260, GSE26712 and GSE32062.

## Author Contributions

XY conceived and designed the study with LL. LL and LC drafted the manuscript and analyzed the data with SY and CL. BL and LP handled the picture and article format. WL, YZ, and JZ reviewed the data. All authors have read and approved the final published manuscript.

## Conflict of Interest

The authors declare that the research was conducted in the absence of any commercial or financial relationships that could be construed as a potential conflict of interest.

## Footnotes

1 https://www.gsea-msigdb.org/gsea/index.jsp
