# Supplementary material for: Construction and Validation of a Novel Glycometabolism-Related Gene Signature Predicting Survival in Patients With Ovarian Cancer
Source: Front Genet. 2020 Nov 12;11:585259. doi: 10.3389/fgene.2020.585259 (PMC7689371; doi:10.3389/fgene.2020.585259)
Supplement: Supplementary file 1 [file Table_1.DOCX]

| ID | futime | fustat |
| --- | --- | --- |
| GSM432220 | 55 | 0 |
| GSM432221 | 1 | 1 |
| GSM432222 | 24 | 0 |
| GSM432223 | 26 | 0 |
| GSM432224 | 33 | 1 |
| GSM432225 | 37 | 0 |
| GSM432226 | 20 | 1 |
| GSM432227 | 47 | 0 |
| GSM432228 | 60 | 0 |
| GSM432229 | 57 | 1 |
| GSM432230 | 48 | 0 |
| GSM432231 | 69 | 0 |
| GSM432232 | 27 | 1 |
| GSM432233 | 69 | 1 |
| GSM432234 | 51 | 1 |
| GSM432235 | 17 | 0 |
| GSM432236 | 17 | 0 |
| GSM432237 | 17 | 0 |
| GSM432238 | 24 | 1 |
| GSM432239 | 52 | 1 |
| GSM432240 | 25 | 1 |
| GSM432242 | 22 | 1 |
| GSM432243 | 53 | 1 |
| GSM432244 | 13 | 1 |
| GSM432245 | 79 | 0 |
| GSM432246 | 74 | 0 |
| GSM432247 | 75 | 0 |
| GSM432248 | 62 | 1 |
| GSM432249 | 64 | 0 |
| GSM432250 | 46 | 0 |
| GSM432251 | 44 | 0 |
| GSM432252 | 28 | 1 |
| GSM432253 | 33 | 0 |
| GSM432254 | 31 | 0 |
| GSM432255 | 28 | 0 |
| GSM432256 | 24 | 0 |
| GSM432257 | 15 | 0 |
| GSM432258 | 15 | 0 |
| GSM432259 | 49 | 0 |
| GSM432260 | 14 | 0 |
| GSM432261 | 13 | 0 |
| GSM432262 | 9 | 0 |
| GSM432263 | 29 | 0 |
| GSM432264 | 26 | 0 |
| GSM432265 | 49 | 0 |
| GSM432266 | 20 | 0 |
| GSM432267 | 47 | 0 |
| GSM432268 | 15 | 1 |
| GSM432269 | 12 | 1 |
| GSM432270 | 26 | 1 |
| GSM432271 | 41 | 1 |
| GSM432272 | 11 | 1 |
| GSM432273 | 47 | 1 |
| GSM432274 | 50 | 0 |
| GSM432275 | 1 | 1 |
| GSM432276 | 72 | 0 |
| GSM432277 | 65 | 0 |
| GSM432278 | 33 | 0 |
| GSM432279 | 29 | 0 |
| GSM432280 | 49 | 1 |
| GSM432281 | 70 | 0 |
| GSM432282 | 15 | 0 |
| GSM432283 | 8 | 1 |
| GSM432284 | 80 | 1 |
| GSM432285 | 11 | 1 |
| GSM432286 | 5 | 1 |
| GSM432287 | 28 | 1 |
| GSM432288 | 25 | 1 |
| GSM432289 | 64 | 1 |
| GSM432290 | 41 | 1 |
| GSM432291 | 61 | 0 |
| GSM432292 | 46 | 0 |
| GSM432293 | 34 | 1 |
| GSM432294 | 17 | 1 |
| GSM432295 | 39 | 0 |
| GSM432296 | 37 | 0 |
| GSM432297 | 33 | 1 |
| GSM432298 | 25 | 0 |
| GSM432299 | 24 | 0 |
| GSM432300 | 23 | 1 |
| GSM432301 | 30 | 0 |
| GSM432302 | 30 | 0 |
| GSM432303 | 29 | 0 |
| GSM432304 | 23 | 0 |
| GSM432305 | 20 | 0 |
| GSM432306 | 32 | 1 |
| GSM432307 | 81 | 0 |
| GSM432308 | 74 | 0 |
| GSM432309 | 26 | 1 |
| GSM432310 | 26 | 0 |
| GSM432311 | 19 | 0 |
| GSM432312 | 22 | 0 |
| GSM432313 | 22 | 0 |
| GSM432314 | 21 | 0 |
| GSM432315 | 42 | 1 |
| GSM432316 | 26 | 1 |
| GSM432317 | 1 | 1 |
| GSM432318 | 27 | 1 |
| GSM432319 | 68 | 1 |
| GSM432320 | 64 | 0 |
| GSM432321 | 52 | 0 |
| GSM432322 | 65 | 0 |
| GSM432323 | 33 | 1 |
| GSM432324 | 51 | 1 |
| GSM432325 | 1 | 1 |
| GSM432326 | 54 | 1 |
| GSM432327 | 51 | 0 |
| GSM432328 | 51 | 0 |
| GSM432329 | 39 | 0 |
| GSM432330 | 28 | 0 |
