# Supplementary material for: Construction and Validation of a Novel Glycometabolism-Related Gene Signature Predicting Survival in Patients With Ovarian Cancer
Source: Front Genet. 2020 Nov 12;11:585259. doi: 10.3389/fgene.2020.585259 (PMC7689371; doi:10.3389/fgene.2020.585259)
Supplement: Supplementary file 2 [file Table_2.DOCX]

| id | futime | fustat | treatment | age | stage |
| --- | --- | --- | --- | --- | --- |
| GSM4153778 | 3.632877 | 0 | 0 | 47 | 4 |
| GSM4153779 | 2.180822 | 1 | 0 | 65 | 4 |
| GSM4153780 | 3.509589 | 0 | 1 | 63 | 3 |
| GSM4153781 | 1.038356 | 1 | 0 | 80 | 3 |
| GSM4153782 | 3.331507 | 0 | 1 | 60 | 3 |
| GSM4153783 | 2.715068 | 0 | 1 | 46 | 3 |
| GSM4153784 | 3.271233 | 0 | 0 | 62 | 3 |
| GSM4153785 | 3 | 0 | 1 | 43 | 3 |
| GSM4153786 | 2.726027 | 0 | 1 | 64 | 3 |
| GSM4153787 | 3.260274 | 0 | 1 | 68 | 3 |
| GSM4153788 | 3.164384 | 0 | 1 | 43 | 3 |
| GSM4153789 | 2.660274 | 0 | 1 | 61 | 4 |
| GSM4153790 | 2.742466 | 0 | 0 | 59 | 2 |
| GSM4153791 | 2.778082 | 0 | 0 | 37 | 3 |
| GSM4153792 | 2.816438 | 0 | 1 | 59 | 3 |
| GSM4153793 | 1.172603 | 1 | 1 | 66 | 3 |
| GSM4153794 | 2.249315 | 0 | 0 | 42 | 3 |
| GSM4153795 | 2.065753 | 0 | 1 | 71 | 4 |
| GSM4153796 | 1.950685 | 1 | 0 | 57 | 3 |
| GSM4153797 | 3.20274 | 0 | 1 | 58 | 3 |
| GSM4153798 | 2.468493 | 0 | 0 | 72 | 3 |
| GSM4153799 | 3.232877 | 0 | 1 | 50 | 3 |
| GSM4153800 | 3.369863 | 0 | 0 | 65 | 3 |
| GSM4153801 | 3.39726 | 0 | 1 | 53 | 3 |
| GSM4153802 | 0.479452 | 0 | 0 | 78 | 4 |
| GSM4153803 | 2.087671 | 1 | 0 | 80 | 4 |
| GSM4153804 | 1.043836 | 1 | 0 | 64 | 3 |
| GSM4153805 | 1.490411 | 1 | 0 | 77 | 3 |
| GSM4153806 | 2.780822 | 0 | 0 | 52 | 1 |
| GSM4153807 | 2.515068 | 0 | 0 | 55 | 3 |
| GSM4153808 | 2.284932 | 0 | 1 | 47 | 3 |
| GSM4153809 | 2.238356 | 0 | 1 | 44 | 1 |
| GSM4153810 | 3.561644 | 0 | 0 | 43 | 3 |
| GSM4153811 | 1.849315 | 1 | 1 | 75 | 3 |
| GSM4153812 | 1.558904 | 1 | 0 | 65 | 3 |
| GSM4153813 | 1.679452 | 0 | 1 | 57 | 3 |
| GSM4153814 | 3.438356 | 0 | 1 | 62 | 3 |
| GSM4153815 | 2.208219 | 1 | 1 | 64 | 3 |
| GSM4153816 | 0.410959 | 1 | 1 | 64 | 3 |
| GSM4153817 | 3.164384 | 0 | 1 | 67 | 1 |
| GSM4153818 | 0.375342 | 0 | 1 | 67 | 2 |
| GSM4153819 | 2.90137 | 1 | 1 | 38 | 3 |
| GSM4153820 | 3.438356 | 0 | 0 | 62 | 4 |
| GSM4153821 | 3.008219 | 0 | 0 | 57 | 2 |
| GSM4153822 | 3.052055 | 0 | 0 | 66 | 3 |
| GSM4153823 | 3.090411 | 0 | 1 | 55 | 1 |
| GSM4153824 | 2.832877 | 0 | 0 | 68 | 3 |
| GSM4153825 | 2.586301 | 0 | 0 | 62 | 3 |
| GSM4153826 | 2.473973 | 0 | 1 | 52 | 3 |
| GSM4153827 | 0.616438 | 1 | 0 | 50 | 3 |
| GSM4153828 | 1.531507 | 1 | 1 | 69 | 3 |
| GSM4153829 | 2.216438 | 0 | 0 | 41 | 2 |
| GSM4153830 | 2.550685 | 1 | 0 | 64 | 4 |
| GSM4153831 | 1.608219 | 1 | 0 | 54 | 3 |
| GSM4153832 | 1.750685 | 0 | 0 | 51 | 1 |
| GSM4153833 | 2.969863 | 0 | 1 | 60 | 3 |
| GSM4153834 | 3.394521 | 0 | 0 | 63 | 3 |
| GSM4153835 | 3.049315 | 1 | 0 | 53 | 3 |
| GSM4153836 | 2.879452 | 0 | 1 | 65 | 3 |
| GSM4153837 | 2.161644 | 0 | 1 | 48 | 3 |
| GSM4153838 | 2 | 1 | 0 | 65 | 3 |
| GSM4153839 | 1.70137 | 1 | 0 | 49 | 3 |
| GSM4153840 | 2.649315 | 1 | 1 | 53 | 3 |
| GSM4153841 | 2.512329 | 0 | 0 | 67 | 3 |
| GSM4153842 | 2.30411 | 0 | 1 | 66 | 3 |
| GSM4153843 | 1.983562 | 0 | 1 | 42 | 2 |
| GSM4153844 | 3.035616 | 0 | 1 | 45 | 3 |
| GSM4153845 | 1.205479 | 1 | 1 | 73 | 3 |
| GSM4153846 | 2.79726 | 0 | 0 | 50 | 1 |
| GSM4153847 | 1.613699 | 0 | 1 | 54 | 1 |
| GSM4153848 | 3.336986 | 0 | 1 | 70 | 3 |
| GSM4153849 | 2.421918 | 1 | 1 | 44 | 3 |
| GSM4153850 | 3.035616 | 0 | 1 | 58 | 3 |
| GSM4153851 | 2.50411 | 0 | 1 | 75 | 2 |
| GSM4153852 | 1.2 | 0 | 0 | 71 | 3 |
| GSM4153853 | 3.131507 | 0 | 1 | 39 | 3 |
| GSM4153854 | 3.394521 | 0 | 0 | 64 | 3 |
| GSM4153855 | 3.353425 | 0 | 0 | 59 | 3 |
| GSM4153856 | 2.254795 | 1 | 1 | 56 | 4 |
| GSM4153857 | 3.241096 | 0 | 1 | 66 | 3 |
| GSM4153858 | 2.753425 | 0 | 1 | 46 | 2 |
| GSM4153859 | 2.016438 | 0 | 1 | 58 | 3 |
| GSM4153860 | 2.857534 | 0 | 1 | 65 | 1 |
| GSM4153861 | 2.589041 | 0 | 0 | 50 | 2 |
| GSM4153862 | 1.263014 | 1 | 1 | 69 | 4 |
| GSM4153863 | 1.630137 | 1 | 1 | 68 | 4 |
| GSM4153864 | 3.167123 | 0 | 1 | 66 | 2 |
| GSM4153865 | 0.79726 | 0 | 0 | 69 | 3 |
| GSM4153866 | 2.463014 | 0 | 1 | 51 | 3 |
| GSM4153867 | 1.89863 | 0 | 1 | 39 | 3 |
| GSM4153868 | 0.624658 | 0 | 1 | 66 | 3 |
| GSM4153869 | 0.564384 | 0 | 0 | 56 | 4 |
| GSM4153870 | 0.832877 | 1 | 1 | 67 | 3 |
| GSM4153871 | 2.835616 | 1 | 1 | 51 | 3 |
| GSM4153872 | 3.271233 | 1 | 1 | 53 | 3 |
| GSM4153873 | 1.032877 | 1 | 1 | 51 | 3 |
| GSM4153874 | 2.093151 | 1 | 1 | 58 | 3 |
| GSM4153875 | 2.871233 | 0 | 1 | 65 | 3 |
| GSM4153876 | 2.561644 | 0 | 0 | 58 | 3 |
| GSM4153877 | 2.583562 | 0 | 1 | 31 | 4 |
| GSM4153878 | 2.145205 | 1 | 1 | 71 | 3 |
| GSM4153879 | 2.517808 | 0 | 0 | 41 | 3 |
| GSM4153880 | 2.457534 | 1 | 0 | 66 | 3 |
| GSM4153881 | 2.567123 | 1 | 1 | 47 | 4 |
| GSM4153882 | 2.457534 | 0 | 1 | 63 | 3 |
| GSM4153883 | 1.857534 | 1 | 0 | 67 | 3 |
| GSM4153884 | 0.364384 | 1 | 0 | 79 | 4 |
| GSM4153885 | 1.6 | 1 | 0 | 53 | 3 |
| GSM4153886 | 1.761644 | 0 | 1 | 70 | 3 |
| GSM4153887 | 1.542466 | 0 | 1 | 76 | 3 |
| GSM4153888 | 2.216438 | 0 | 1 | 44 | 3 |
| GSM4153889 | 2.016438 | 0 | 1 | 47 | 2 |
| GSM4153890 | 0.961644 | 1 | 0 | 50 | 4 |
| GSM4153891 | 1.805479 | 0 | 1 | 57 | 3 |
| GSM4153892 | 1.778082 | 0 | 0 | 52 | 3 |
| GSM4153893 | 2.742466 | 0 | 1 | 51 | 3 |
| GSM4153894 | 3.145205 | 0 | 0 | 56 | 3 |
| GSM4153895 | 3.060274 | 0 | 1 | 53 | 4 |
| GSM4153896 | 2.189041 | 1 | 1 | 46 | 4 |
| GSM4153897 | 2.049315 | 0 | 0 | 51 | 4 |
| GSM4153898 | 3.09589 | 0 | 0 | 66 | 3 |
| GSM4153899 | 3.073973 | 0 | 1 | 56 | 3 |
| GSM4153900 | 2.991781 | 0 | 0 | 57 | 1 |
| GSM4153901 | 1.827397 | 1 | 0 | 44 | 3 |
| GSM4153902 | 0.386301 | 1 | 1 | 71 | 3 |
| GSM4153903 | 2.024658 | 0 | 0 | 21 | 3 |
| GSM4153904 | 3.230137 | 0 | 0 | 37 | 3 |
| GSM4153905 | 2.347945 | 0 | 1 | 70 | 3 |
| GSM4153906 | 2.271233 | 1 | 1 | 59 | 1 |
| GSM4153907 | 1.687671 | 1 | 1 | 26 | 4 |
| GSM4153908 | 1.742466 | 0 | 1 | 67 | 3 |
| GSM4153909 | 1.649315 | 0 | 0 | 68 | 3 |
| GSM4153910 | 2.6 | 1 | 0 | 68 | 3 |
| GSM4153911 | 1.649315 | 1 | 1 | 63 | 3 |
| GSM4153912 | 3.224658 | 0 | 0 | 39 | 4 |
| GSM4153913 | 2.638356 | 0 | 0 | 62 | 3 |
| GSM4153914 | 2.386301 | 0 | 1 | 56 | 3 |
| GSM4153915 | 2.235616 | 0 | 1 | 43 | 1 |
| GSM4153916 | 2.150685 | 0 | 0 | 51 | 4 |
| GSM4153917 | 2.060274 | 0 | 1 | 73 | 3 |
| GSM4153918 | 1.841096 | 0 | 0 | 62 | 2 |
| GSM4153919 | 2.145205 | 1 | 1 | 69 | 3 |
| GSM4153920 | 2.906849 | 0 | 0 | 70 | 2 |
| GSM4153921 | 1.980822 | 1 | 1 | 66 | 3 |
| GSM4153922 | 2.789041 | 0 | 1 | 59 | 3 |
| GSM4153923 | 3.054795 | 0 | 0 | 57 | 3 |
| GSM4153924 | 3.153425 | 0 | 1 | 44 | 4 |
| GSM4153925 | 2.216438 | 1 | 1 | 50 | 4 |
| GSM4153926 | 2.580822 | 0 | 0 | 61 | 3 |
| GSM4153927 | 2.265753 | 0 | 0 | 73 | 2 |
| GSM4153928 | 1.986301 | 0 | 1 | 36 | 3 |
| GSM4153929 | 1.706849 | 1 | 0 | 63 | 3 |
| GSM4153930 | 1.421918 | 0 | 0 | 68 | 3 |
| GSM4153931 | 2.887671 | 0 | 1 | 45 | 3 |
| GSM4153932 | 0.479452 | 0 | 1 | 71 | 3 |
| GSM4153933 | 2.476712 | 0 | 1 | 57 | 3 |
| GSM4153934 | 2.249315 | 0 | 1 | 68 | 3 |
| GSM4153935 | 1.986301 | 0 | 1 | 57 | 2 |
| GSM4153936 | 1.978082 | 0 | 0 | 65 | 3 |
| GSM4153937 | 1.487671 | 0 | 1 | 52 | 3 |
| GSM4153938 | 3.021918 | 0 | 0 | 49 | 2 |
| GSM4153939 | 2.956164 | 0 | 0 | 51 | 3 |
| GSM4153940 | 2.947945 | 0 | 0 | 52 | 3 |
| GSM4153941 | 1.682192 | 1 | 0 | 66 | 4 |
| GSM4153942 | 1.978082 | 0 | 0 | 44 | 3 |
| GSM4153943 | 2.876712 | 0 | 0 | 53 | 3 |
| GSM4153944 | 1.769863 | 1 | 0 | 68 | 3 |
| GSM4153945 | 2.09589 | 0 | 1 | 63 | 3 |
| GSM4153946 | 1.578082 | 1 | 0 | 65 | 3 |
| GSM4153947 | 2 | 0 | 1 | 71 | 3 |
| GSM4153948 | 1.772603 | 0 | 1 | 72 | 3 |
| GSM4153949 | 2.616438 | 1 | 1 | 64 | 4 |
| GSM4153950 | 2.328767 | 1 | 1 | 47 | 3 |
| GSM4153951 | 1.90137 | 0 | 0 | 59 | 3 |
| GSM4153952 | 0.980822 | 1 | 1 | 34 | 4 |
| GSM4153953 | 2.139726 | 1 | 0 | 66 | 3 |
| GSM4153954 | 2.09589 | 0 | 0 | 43 | 3 |
| GSM4153955 | 2.065753 | 0 | 1 | 69 | 3 |
| GSM4153956 | 0.336986 | 1 | 0 | 47 | 1 |
| GSM4153957 | 2.813699 | 1 | 0 | 69 | 3 |
| GSM4153958 | 2.690411 | 0 | 1 | 61 | 4 |
| GSM4153959 | 0.567123 | 0 | 0 | 65 | 4 |
| GSM4153960 | 1.076712 | 1 | 0 | 56 | 4 |
| GSM4153961 | 1.783562 | 0 | 1 | 48 | 3 |
| GSM4153962 | 1.569863 | 0 | 0 | 70 | 3 |
| GSM4153963 | 2.745205 | 0 | 0 | 59 | 3 |
| GSM4153964 | 2.183562 | 0 | 0 | 48 | 3 |
| GSM4153965 | 3.115068 | 0 | 0 | 49 | 3 |
| GSM4153966 | 2.717808 | 0 | 1 | 37 | 2 |
| GSM4153967 | 3.112329 | 0 | 1 | 69 | 3 |
| GSM4153968 | 2.534247 | 0 | 1 | 72 | 3 |
| GSM4153969 | 2.126027 | 1 | 0 | 75 | 3 |
| GSM4153970 | 2.29589 | 1 | 0 | 66 | 3 |
| GSM4153971 | 2.30411 | 0 | 1 | 70 | 4 |
| GSM4153972 | 2.819178 | 0 | 1 | 49 | 2 |
| GSM4153973 | 3.005479 | 0 | 1 | 60 | 3 |
| GSM4153974 | 2.50411 | 0 | 0 | 59 | 3 |
| GSM4153975 | 0.019178 | 0 | 1 | 73 | 4 |
| GSM4153976 | 1.969863 | 0 | 0 | 70 | 2 |
| GSM4153977 | 1.838356 | 0 | 1 | 68 | 3 |
| GSM4153978 | 2.652055 | 1 | 0 | 42 | 3 |
| GSM4153979 | 2.953425 | 0 | 0 | 53 | 3 |
| GSM4153980 | 2.860274 | 0 | 1 | 64 | 3 |
| GSM4153981 | 2.736986 | 0 | 0 | 65 | 4 |
| GSM4153982 | 2.652055 | 0 | 1 | 53 | 2 |
| GSM4153983 | 0.134247 | 0 | 0 | 23 | 3 |
| GSM4153984 | 0.978082 | 1 | 0 | 65 | 4 |
| GSM4153985 | 1.983562 | 0 | 1 | 50 | 3 |
| GSM4153986 | 2.008219 | 0 | 0 | 60 | 1 |
| GSM4153987 | 2.841096 | 0 | 0 | 41 | 2 |
| GSM4153988 | 1.813699 | 1 | 0 | 55 | 3 |
| GSM4153989 | 2.791781 | 0 | 1 | 69 | 2 |
| GSM4153990 | 3.060274 | 0 | 0 | 57 | 3 |
| GSM4153991 | 2.323288 | 0 | 1 | 63 | 4 |
| GSM4153992 | 2.079452 | 0 | 1 | 68 | 4 |
| GSM4153993 | 2.819178 | 0 | 0 | 48 | 3 |
| GSM4153994 | 2.835616 | 0 | 0 | 61 | 3 |
| GSM4153995 | 2.410959 | 0 | 1 | 58 | 3 |
| GSM4153996 | 2.115068 | 0 | 0 | 46 | 3 |
| GSM4153997 | 1.90137 | 0 | 0 | 62 | 3 |
| GSM4153998 | 1.378082 | 1 | 0 | 73 | 3 |
| GSM4153999 | 1.789041 | 0 | 1 | 65 | 3 |
| GSM4154000 | 0.786301 | 1 | 0 | 64 | 4 |
| GSM4154001 | 2.942466 | 0 | 1 | 43 | 2 |
| GSM4154002 | 2.479452 | 0 | 0 | 64 | 3 |
| GSM4154003 | 0.958904 | 1 | 1 | 48 | 4 |
| GSM4154004 | 1.832877 | 1 | 1 | 65 | 3 |
| GSM4154005 | 1.627397 | 1 | 1 | 64 | 3 |
| GSM4154006 | 2.657534 | 0 | 0 | 58 | 4 |
| GSM4154007 | 1.021918 | 1 | 0 | 57 | 4 |
| GSM4154008 | 2.252055 | 0 | 1 | 70 | 4 |
| GSM4154009 | 2.172603 | 0 | 1 | 63 | 3 |
| GSM4154010 | 1.627397 | 1 | 1 | 35 | 3 |
| GSM4154011 | 0.747945 | 1 | 0 | 62 | 3 |
| GSM4154012 | 1.715068 | 0 | 1 | 77 | 3 |
| GSM4154013 | 2.764384 | 0 | 1 | 46 | 3 |
| GSM4154014 | 2.758904 | 0 | 1 | 58 | 3 |
| GSM4154015 | 2.821918 | 0 | 1 | 58 | 4 |
| GSM4154016 | 2.734247 | 0 | 0 | 57 | 1 |
| GSM4154017 | 0.926027 | 1 | 0 | 75 | 3 |
| GSM4154018 | 2.073973 | 0 | 1 | 57 | 2 |
| GSM4154019 | 1.769863 | 0 | 0 | 57 | 2 |
| GSM4154020 | 1.917808 | 0 | 1 | 58 | 4 |
| GSM4154021 | 1.747945 | 0 | 1 | 63 | 3 |
| GSM4154022 | 2.824658 | 0 | 1 | 62 | 4 |
| GSM4154023 | 2.690411 | 0 | 1 | 60 | 3 |
| GSM4154024 | 2.764384 | 0 | 0 | 59 | 3 |
| GSM4154025 | 1.484932 | 1 | 0 | 67 | 3 |
| GSM4154026 | 1.350685 | 0 | 1 | 68 | 3 |
| GSM4154027 | 2.690411 | 0 | 1 | 55 | 3 |
| GSM4154028 | 2.443836 | 0 | 0 | 66 | 3 |
| GSM4154029 | 2.572603 | 0 | 1 | 42 | 2 |
| GSM4154030 | 2.293151 | 0 | 0 | 59 | 4 |
| GSM4154031 | 2.460274 | 0 | 0 | 43 | 3 |
| GSM4154032 | 2.271233 | 0 | 0 | 56 | 3 |
| GSM4154033 | 2.254795 | 0 | 1 | 46 | 3 |
| GSM4154034 | 2.268493 | 0 | 0 | 38 | 3 |
| GSM4154035 | 2.232877 | 0 | 0 | 60 | 4 |
| GSM4154036 | 0.452055 | 0 | 0 | 64 | 1 |
| GSM4154037 | 2.076712 | 0 | 0 | 70 | 1 |
| GSM4154038 | 2.161644 | 0 | 0 | 55 | 3 |
| GSM4154039 | 2.131507 | 0 | 1 | 58 | 3 |
| GSM4154040 | 2.180822 | 0 | 1 | 63 | 3 |
| GSM4154041 | 1.742466 | 0 | 0 | 38 | 4 |
| GSM4154042 | 1.950685 | 0 | 1 | 62 | 3 |
| GSM4154043 | 1.808219 | 0 | 0 | 54 | 3 |
| GSM4154044 | 1.578082 | 0 | 1 | 57 | 3 |
| GSM4154045 | 0.687671 | 1 | 0 | 61 | 3 |
| GSM4154046 | 2.263014 | 0 | 1 | 66 | 3 |
| GSM4154047 | 2.210959 | 0 | 0 | 58 | 3 |
| GSM4154048 | 0.054795 | 0 | 0 | 21 | 3 |
| GSM4154049 | 2.232877 | 0 | 1 | 63 | 3 |
| GSM4154050 | 2.534247 | 0 | 1 | 61 | 3 |
| GSM4154051 | 2.290411 | 0 | 1 | 27 | 3 |
| GSM4154052 | 2.479452 | 0 | 1 | 27 | 3 |
| GSM4154053 | 2.339726 | 0 | 1 | 49 | 3 |
| GSM4154054 | 1.435616 | 1 | 0 | 70 | 3 |
| GSM4154055 | 2.556164 | 0 | 1 | 54 | 3 |
| GSM4154056 | 1.854795 | 0 | 1 | 66 | 3 |
| GSM4154057 | 2.564384 | 0 | 0 | 64 | 1 |
| GSM4154058 | 2.260274 | 0 | 1 | 56 | 2 |
| GSM4154059 | 0.676712 | 1 | 1 | 68 | 3 |
| GSM4154060 | 1.767123 | 0 | 1 | 55 | 3 |
| GSM4154061 | 1.767123 | 0 | 1 | 70 | 1 |
| GSM4154062 | 0.493151 | 1 | 1 | 76 | 3 |
| GSM4154063 | 2.054795 | 0 | 1 | 68 | 4 |
| GSM4154064 | 2.312329 | 0 | 0 | 37 | 4 |
| GSM4154065 | 1.693151 | 0 | 0 | 66 | 3 |
| GSM4154066 | 0.983562 | 1 | 0 | 48 | 4 |
| GSM4154067 | 1.10411 | 1 | 1 | 46 | 3 |
| GSM4154068 | 2.635616 | 0 | 0 | 45 | 3 |
| GSM4154069 | 2.123288 | 0 | 0 | 65 | 3 |
| GSM4154070 | 1.564384 | 0 | 0 | 72 | 3 |
| GSM4154071 | 1.564384 | 1 | 0 | 67 | 3 |
| GSM4154072 | 0.00274 | 0 | 0 | 49 | 2 |
| GSM4154073 | 2.49863 | 0 | 0 | 47 | 3 |
| GSM4154074 | 2.169863 | 1 | 0 | 49 | 4 |
| GSM4154075 | 1.684932 | 1 | 1 | 64 | 3 |
| GSM4154076 | 2.131507 | 0 | 1 | 49 | 2 |
| GSM4154077 | 2.191781 | 0 | 1 | 65 | 3 |
| GSM4154078 | 2.569863 | 0 | 1 | 52 | 3 |
| GSM4154079 | 2.580822 | 0 | 0 | 60 | 3 |
| GSM4154080 | 0.928767 | 1 | 0 | 54 | 3 |
| GSM4154081 | 1.517808 | 0 | 0 | 45 | 3 |
| GSM4154082 | 1.479452 | 0 | 0 | 57 | 3 |
| GSM4154083 | 0.950685 | 1 | 1 | 69 | 3 |
| GSM4154084 | 2.186301 | 0 | 0 | 56 | 3 |
| GSM4154085 | 2.358904 | 0 | 1 | 48 | 3 |
| GSM4154086 | 1.808219 | 0 | 1 | 60 | 1 |
| GSM4154087 | 2.109589 | 0 | 1 | 68 | 3 |
| GSM4154088 | 1.452055 | 1 | 1 | 61 | 3 |
| GSM4154089 | 0.591781 | 1 | 0 | 66 | 3 |
| GSM4154090 | 1.947945 | 0 | 1 | 44 | 3 |
| GSM4154091 | 2.342466 | 0 | 0 | 53 | 3 |
| GSM4154092 | 2.235616 | 0 | 1 | 73 | 2 |
| GSM4154093 | 2.249315 | 1 | 0 | 48 | 2 |
| GSM4154094 | 2.268493 | 0 | 1 | 47 | 3 |
| GSM4154095 | 2.147945 | 0 | 1 | 68 | 4 |
| GSM4154096 | 2.032877 | 0 | 1 | 55 | 3 |
| GSM4154097 | 2 | 0 | 1 | 53 | 3 |
| GSM4154098 | 1.580822 | 0 | 1 | 45 | 4 |
| GSM4154099 | 0.29863 | 0 | 1 | 49 | 3 |
| GSM4154100 | 0.90411 | 0 | 0 | 42 | 3 |
| GSM4154101 | 1.813699 | 0 | 1 | 57 | 1 |
| GSM4154102 | 1.978082 | 0 | 0 | 56 | 3 |
| GSM4154103 | 0.328767 | 0 | 0 | 69 | 4 |
| GSM4154104 | 2.030137 | 0 | 1 | 47 | 4 |
| GSM4154105 | 1.756164 | 0 | 0 | 56 | 3 |
| GSM4154106 | 2.112329 | 0 | 0 | 62 | 4 |
| GSM4154107 | 1.594521 | 0 | 0 | 70 | 3 |
| GSM4154108 | 1.99726 | 0 | 1 | 34 | 3 |
| GSM4154109 | 1.750685 | 0 | 1 | 65 | 3 |
| GSM4154110 | 2 | 0 | 1 | 54 | 3 |
| GSM4154111 | 2.123288 | 0 | 1 | 62 | 3 |
| GSM4154112 | 0.758904 | 1 | 0 | 25 | 3 |
| GSM4154113 | 2.323288 | 0 | 0 | 45 | 4 |
| GSM4154114 | 2.109589 | 0 | 1 | 69 | 4 |
| GSM4154115 | 0.476712 | 0 | 0 | 73 | 3 |
| GSM4154116 | 1.726027 | 1 | 0 | 71 | 3 |
| GSM4154117 | 2.027397 | 0 | 0 | 49 | 4 |
| GSM4154118 | 1.687671 | 0 | 0 | 44 | 3 |
| GSM4154119 | 1.958904 | 0 | 0 | 46 | 4 |
| GSM4154120 | 1.526027 | 0 | 1 | 46 | 3 |
| GSM4154121 | 1.380822 | 1 | 1 | 68 | 3 |
| GSM4154122 | 1.978082 | 0 | 0 | 67 | 3 |
| GSM4154123 | 1.849315 | 0 | 1 | 39 | 3 |
| GSM4154124 | 1.89589 | 0 | 0 | 63 | 3 |
| GSM4154125 | 1.767123 | 0 | 0 | 46 | 3 |
| GSM4154126 | 1.712329 | 0 | 0 | 50 | 3 |
| GSM4154127 | 2.271233 | 0 | 1 | 78 | 3 |
| GSM4154128 | 2.038356 | 0 | 0 | 53 | 3 |
| GSM4154129 | 1.065753 | 1 | 1 | 53 | 3 |
| GSM4154130 | 0.561644 | 0 | 0 | 53 | 3 |
| GSM4154131 | 1.841096 | 0 | 1 | 44 | 3 |
| GSM4154132 | 1.589041 | 1 | 0 | 65 | 3 |
| GSM4154133 | 0.794521 | 1 | 0 | 71 | 3 |
| GSM4154134 | 1.424658 | 1 | 1 | 80 | 4 |
| GSM4154135 | 2.09589 | 0 | 0 | 70 | 3 |
| GSM4154136 | 0.643836 | 1 | 0 | 46 | 3 |
| GSM4154137 | 2.167123 | 0 | 1 | 77 | 3 |
| GSM4154138 | 0.923288 | 0 | 0 | 59 | 3 |
| GSM4154139 | 1.671233 | 0 | 1 | 70 | 3 |
| GSM4154140 | 1.849315 | 0 | 1 | 62 | 3 |
| GSM4154141 | 1.906849 | 0 | 1 | 47 | 3 |
| GSM4154142 | 1.364384 | 0 | 0 | 69 | 3 |
| GSM4154143 | 2.09589 | 0 | 1 | 61 | 4 |
| GSM4154144 | 2.087671 | 0 | 1 | 60 | 3 |
| GSM4154145 | 1.791781 | 0 | 1 | 54 | 3 |
| GSM4154146 | 1.857534 | 0 | 1 | 64 | 3 |
| GSM4154147 | 1.624658 | 0 | 1 | 66 | 3 |
| GSM4154148 | 0.049315 | 0 | 0 | 43 | 3 |
| GSM4154149 | 1.712329 | 0 | 1 | 69 | 3 |
| GSM4154150 | 1.80274 | 0 | 0 | 57 | 2 |
| GSM4154151 | 1.30137 | 1 | 1 | 54 | 4 |
| GSM4154152 | 1.767123 | 0 | 0 | 70 | 3 |
| GSM4154153 | 1.747945 | 0 | 0 | 56 | 3 |
| GSM4154154 | 1.767123 | 0 | 0 | 37 | 3 |
| GSM4154155 | 1.756164 | 0 | 0 | 60 | 3 |
| GSM4154156 | 1.753425 | 0 | 1 | 58 | 3 |
| GSM4154157 | 1.561644 | 0 | 0 | 49 | 3 |
