# Supplementary material for: Construction and Validation of a Novel Glycometabolism-Related Gene Signature Predicting Survival in Patients With Ovarian Cancer
Source: Front Genet. 2020 Nov 12;11:585259. doi: 10.3389/fgene.2020.585259 (PMC7689371; doi:10.3389/fgene.2020.585259)
Supplement: Supplementary file 3 [file Table_3.DOCX]

| id | coef | HR |
| --- | --- | --- |
| B3GAT3 | -0.13605 | 0.872797 |
| COL5A1 | 0.3101 | 1.363562 |
| FAM162A | 0.424815 | 1.529308 |
| IDUA | -0.09117 | 0.912864 |
| PPP2R1A | -0.2105 | 0.810176 |
