# Supplementary material for: Construction and Validation of a Novel Glycometabolism-Related Gene Signature Predicting Survival in Patients With Ovarian Cancer
Source: Front Genet. 2020 Nov 12;11:585259. doi: 10.3389/fgene.2020.585259 (PMC7689371; doi:10.3389/fgene.2020.585259)
Supplement: Supplementary file 4 [file Table_4.DOCX]

| id | futime | fustat | B3GAT3 | COL5A1 | FAM162A | IDUA | PPP2R1A | riskScore | risk |
| --- | --- | --- | --- | --- | --- | --- | --- | --- | --- |
| GSM4153778 | 3.632877 | 0 | 8.441975 | 8.292992 | 11.37664 | 8.795782 | 12.2991 | 0.806901 | low |
| GSM4153779 | 2.180822 | 1 | 8.34796 | 9.629237 | 11.27218 | 10.54751 | 12.30277 | 1.007784 | low |
| GSM4153780 | 3.509589 | 0 | 9.853646 | 11.98213 | 11.00438 | 11.28303 | 12.69961 | 1.307579 | high |
| GSM4153781 | 1.038356 | 1 | 8.933277 | 10.72622 | 11.51971 | 9.968408 | 0 | 20.41003 | high |
| GSM4153782 | 3.331507 | 0 | 8.892895 | 11.84301 | 11.04776 | 10.2606 | 12.28453 | 1.741536 | high |
| GSM4153783 | 2.715068 | 0 | 9.782801 | 10.0775 | 11.52074 | 11.72908 | 12.5064 | 0.910784 | low |
| GSM4153784 | 3.271233 | 0 | 11.36993 | 10.29221 | 10.12299 | 11.84087 | 12.33516 | 0.444534 | low |
| GSM4153785 | 3 | 0 | 8.303508 | 11.52797 | 10.64634 | 10.11945 | 11.55318 | 1.704975 | high |
| GSM4153786 | 2.726027 | 0 | 8.352653 | 7.828194 | 10.88633 | 11.37734 | 12.55332 | 0.430117 | low |
| GSM4153787 | 3.260274 | 0 | 9.570616 | 9.294237 | 10.45662 | 10.19606 | 12.26303 | 0.566357 | low |
| GSM4153788 | 3.164384 | 0 | 8.367566 | 10.01043 | 11.14245 | 10.67515 | 12.58843 | 0.99642 | low |
| GSM4153789 | 2.660274 | 0 | 10.40148 | 7.216272 | 9.532556 | 12.29012 | 11.47493 | 0.174904 | low |
| GSM4153790 | 2.742466 | 0 | 8.661447 | 10.25185 | 10.69559 | 10.66893 | 12.20736 | 0.925191 | low |
| GSM4153791 | 2.778082 | 0 | 9.962518 | 11.02606 | 10.49423 | 11.48663 | 12.37533 | 0.810477 | low |
| GSM4153792 | 2.816438 | 0 | 9.246471 | 12.05981 | 11.94159 | 11.03234 | 12.74624 | 2.194722 | high |
| GSM4153793 | 1.172603 | 1 | 5.683117 | 11.12885 | 11.21304 | 12.22526 | 12.71424 | 1.76941 | high |
| GSM4153794 | 2.249315 | 0 | 10.77597 | 9.865271 | 9.105154 | 10.45364 | 12.3818 | 0.307878 | low |
| GSM4153795 | 2.065753 | 0 | 9.847971 | 8.849779 | 12.40752 | 8.402992 | 12.61763 | 1.189468 | high |
| GSM4153796 | 1.950685 | 1 | 8.526991 | 9.600673 | 11.28904 | 10.10005 | 12.82487 | 0.916302 | low |
| GSM4153797 | 3.20274 | 0 | 7.672246 | 6.631456 | 11.23 | 10.55788 | 12.55107 | 0.40614 | low |
| GSM4153798 | 2.468493 | 0 | 10.48737 | 10.09976 | 11.12017 | 11.54183 | 12.80055 | 0.672051 | low |
| GSM4153799 | 3.232877 | 0 | 5.399993 | 8.989218 | 10.01397 | 10.4955 | 12.38438 | 0.714207 | low |
| GSM4153800 | 3.369863 | 0 | 7.850519 | 9.886933 | 11.14922 | 10.5901 | 12.54186 | 1.050108 | high |
| GSM4153801 | 3.39726 | 0 | 10.5687 | 10.35606 | 10.93004 | 10.47194 | 11.91959 | 0.880918 | low |
| GSM4153802 | 0.479452 | 0 | 9.208887 | 9.670836 | 12.44982 | 8.713473 | 12.33933 | 1.756481 | high |
| GSM4153803 | 2.087671 | 1 | 7.355166 | 11.38795 | 10.27639 | 11.52591 | 12.50457 | 1.142816 | high |
| GSM4153804 | 1.043836 | 1 | 9.329479 | 10.54977 | 10.97456 | 10.80229 | 11.77318 | 1.129181 | high |
| GSM4153805 | 1.490411 | 1 | 5.462764 | 10.60477 | 10.9987 | 10.85663 | 13.85414 | 1.260962 | high |
| GSM4153806 | 2.780822 | 0 | 7.558657 | 10.63285 | 10.19042 | 11.36688 | 12.73359 | 0.819895 | low |
| GSM4153807 | 2.515068 | 0 | 8.301854 | 10.52441 | 10.62227 | 11.35842 | 12.50946 | 0.903108 | low |
| GSM4153808 | 2.284932 | 0 | 10.12008 | 10.15167 | 10.8181 | 8.987374 | 12.83628 | 0.791115 | low |
| GSM4153809 | 2.238356 | 0 | 9.552866 | 10.884 | 10.05841 | 10.37851 | 12.72922 | 0.699728 | low |
| GSM4153810 | 3.561644 | 0 | 10.36518 | 9.85331 | 10.90735 | 11.61166 | 11.83945 | 0.703506 | low |
| GSM4153811 | 1.849315 | 1 | 7.717924 | 10.19637 | 10.74677 | 9.448363 | 12.55697 | 1.097282 | high |
| GSM4153812 | 1.558904 | 1 | 10.66601 | 10.54357 | 10.43669 | 5.360739 | 11.90475 | 1.194391 | high |
| GSM4153813 | 1.679452 | 0 | 9.828855 | 9.449045 | 10.92599 | 11.30215 | 12.39765 | 0.615425 | low |
| GSM4153814 | 3.438356 | 0 | 6.317894 | 11.83406 | 10.84727 | 10.23952 | 12.26386 | 2.278292 | high |
| GSM4153815 | 2.208219 | 1 | 8.739251 | 12.33233 | 11.23974 | 10.24725 | 12.74717 | 2.039677 | high |
| GSM4153816 | 0.410959 | 1 | 8.967079 | 10.19107 | 10.98144 | 9.95633 | 12.39596 | 1.008553 | low |
| GSM4153817 | 3.164384 | 0 | 7.742389 | 6.58991 | 11.03169 | 9.805108 | 12.63551 | 0.384091 | low |
| GSM4153818 | 0.375342 | 0 | 9.297005 | 11.14477 | 11.51 | 11.12183 | 12.77402 | 1.347258 | high |
| GSM4153819 | 2.90137 | 1 | 9.591852 | 10.40839 | 11.55149 | 7.460039 | 12.85723 | 1.438433 | high |
| GSM4153820 | 3.438356 | 0 | 8.831408 | 9.277487 | 10.95703 | 11.04443 | 12.16225 | 0.728548 | low |
| GSM4153821 | 3.008219 | 0 | 5.407619 | 8.103948 | 9.937793 | 10.78686 | 12.19097 | 0.532404 | low |
| GSM4153822 | 3.052055 | 0 | 10.11844 | 8.645115 | 11.23099 | 10.66174 | 12.22212 | 0.57739 | low |
| GSM4153823 | 3.090411 | 0 | 5.944253 | 10.49981 | 10.02012 | 12.37492 | 11.77743 | 1.016979 | low |
| GSM4153824 | 2.832877 | 0 | 10.13878 | 9.363066 | 11.41142 | 9.440636 | 12.21584 | 0.869299 | low |
| GSM4153825 | 2.586301 | 0 | 10.73446 | 8.385077 | 10.53659 | 12.27339 | 12.17337 | 0.318114 | low |
| GSM4153826 | 2.473973 | 0 | 10.91794 | 10.29191 | 10.67676 | 12.01205 | 11.90549 | 0.644529 | low |
| GSM4153827 | 0.616438 | 1 | 5.483747 | 12.33973 | 10.56443 | 5.716724 | 12.6583 | 3.679853 | high |
| GSM4153828 | 1.531507 | 1 | 7.359721 | 12.01755 | 10.76221 | 7.910634 | 12.78531 | 2.236701 | high |
| GSM4153829 | 2.216438 | 0 | 9.225543 | 9.397151 | 9.708766 | 11.69777 | 12.31249 | 0.384932 | low |
| GSM4153830 | 2.550685 | 1 | 9.192344 | 10.79568 | 11.25634 | 10.98572 | 12.49112 | 1.183243 | high |
| GSM4153831 | 1.608219 | 1 | 5.531541 | 12.14331 | 10.79863 | 8.560889 | 12.64478 | 2.940265 | high |
| GSM4153832 | 1.750685 | 0 | 9.351262 | 11.67495 | 10.96433 | 10.16498 | 12.57364 | 1.422911 | high |
| GSM4153833 | 2.969863 | 0 | 9.338946 | 11.54146 | 10.69089 | 11.52283 | 12.2012 | 1.163499 | high |
| GSM4153834 | 3.394521 | 0 | 8.950818 | 11.65368 | 10.99363 | 10.87304 | 12.88641 | 1.326632 | high |
| GSM4153835 | 3.049315 | 1 | 10.06167 | 10.29093 | 10.87438 | 5.477318 | 12.46392 | 1.270128 | high |
| GSM4153836 | 2.879452 | 0 | 9.711517 | 9.243648 | 10.4861 | 11.44563 | 12.52705 | 0.467497 | low |
| GSM4153837 | 2.161644 | 0 | 9.490403 | 10.73542 | 10.16626 | 8.879153 | 12.62087 | 0.827519 | low |
| GSM4153838 | 2 | 1 | 7.332273 | 11.16311 | 10.93826 | 12.25991 | 12.24286 | 1.399668 | high |
| GSM4153839 | 1.70137 | 1 | 6.458986 | 11.36958 | 11.25197 | 9.942001 | 12.80459 | 2.107322 | high |
| GSM4153840 | 2.649315 | 1 | 8.945219 | 11.461 | 10.86092 | 10.50542 | 12.21393 | 1.40826 | high |
| GSM4153841 | 2.512329 | 0 | 7.779168 | 11.72165 | 10.43184 | 5.185727 | 12.8124 | 2.135194 | high |
| GSM4153842 | 2.30411 | 0 | 6.277761 | 11.34653 | 11.27014 | 10.12695 | 12.55784 | 2.238314 | high |
| GSM4153843 | 1.983562 | 0 | 5.753543 | 11.10511 | 10.27994 | 9.686398 | 12.60472 | 1.509572 | high |
| GSM4153844 | 3.035616 | 0 | 8.416361 | 11.2151 | 12.04846 | 8.235925 | 12.90789 | 2.467895 | high |
| GSM4153845 | 1.205479 | 1 | 8.658748 | 9.396471 | 11.06805 | 12.02838 | 12.08114 | 0.754439 | low |
| GSM4153846 | 2.79726 | 0 | 8.474718 | 9.504726 | 11.46985 | 11.53008 | 12.39976 | 0.928557 | low |
| GSM4153847 | 1.613699 | 0 | 5.461999 | 9.37352 | 10.284 | 11.45177 | 12.11345 | 0.868244 | low |
| GSM4153848 | 3.336986 | 0 | 10.51923 | 9.538664 | 10.49617 | 11.70236 | 12.08077 | 0.494626 | low |
| GSM4153849 | 2.421918 | 1 | 8.430218 | 11.77033 | 11.36454 | 10.29449 | 12.33356 | 2.046927 | high |
| GSM4153850 | 3.035616 | 0 | 9.860906 | 11.23472 | 11.13051 | 12.40343 | 12.42884 | 1.044826 | high |
| GSM4153851 | 2.50411 | 0 | 10.02935 | 9.515101 | 10.23599 | 5.551357 | 12.76847 | 0.712395 | low |
| GSM4153852 | 1.2 | 0 | 8.133236 | 7.238178 | 9.547762 | 7.816987 | 12.39679 | 0.29882 | low |
| GSM4153853 | 3.131507 | 0 | 8.959377 | 10.70923 | 9.605056 | 11.07407 | 12.84087 | 0.543339 | low |
| GSM4153854 | 3.394521 | 0 | 9.723246 | 10.54945 | 9.968408 | 10.84958 | 12.68447 | 0.573643 | low |
| GSM4153855 | 3.353425 | 0 | 9.611191 | 8.830318 | 10.86092 | 10.97653 | 12.89526 | 0.472177 | low |
| GSM4153856 | 2.254795 | 1 | 10.282 | 10.36938 | 11.40013 | 5.579869 | 12.04699 | 1.707844 | high |
| GSM4153857 | 3.241096 | 0 | 8.110265 | 10.2203 | 10.91099 | 10.70146 | 12.4955 | 1.015456 | low |
| GSM4153858 | 2.753425 | 0 | 10.49781 | 10.40939 | 10.64667 | 11.9109 | 12.08038 | 0.679756 | low |
| GSM4153859 | 2.016438 | 0 | 7.984412 | 11.23132 | 10.82572 | 9.551509 | 12.05759 | 1.659928 | high |
| GSM4153860 | 2.857534 | 0 | 9.871536 | 9.841378 | 11.13442 | 11.59036 | 13.10699 | 0.633411 | low |
| GSM4153861 | 2.589041 | 0 | 7.540386 | 10.36938 | 10.7351 | 11.23438 | 12.76412 | 0.960066 | low |
| GSM4153862 | 1.263014 | 1 | 8.890644 | 10.93561 | 12.00714 | 10.21512 | 13.10531 | 1.669654 | high |
| GSM4153863 | 1.630137 | 1 | 9.108692 | 11.03272 | 11.27421 | 11.18002 | 11.97376 | 1.421808 | high |
| GSM4153864 | 3.167123 | 0 | 9.347875 | 8.208266 | 11.57205 | 12.1013 | 12.85009 | 0.497375 | low |
| GSM4153865 | 0.79726 | 0 | 10.01236 | 9.870902 | 10.18129 | 11.15907 | 12.41775 | 0.503015 | low |
| GSM4153866 | 2.463014 | 0 | 7.966187 | 7.369659 | 10.44781 | 10.78329 | 12.83577 | 0.324686 | low |
| GSM4153867 | 1.89863 | 0 | 9.428164 | 6.348373 | 10.52843 | 10.23889 | 12.37372 | 0.232384 | low |
| GSM4153868 | 0.624658 | 0 | 7.825455 | 11.36622 | 10.26433 | 12.24248 | 12.31532 | 1.032684 | low |
| GSM4153869 | 0.564384 | 0 | 9.078852 | 10.45953 | 11.80592 | 9.742468 | 12.61441 | 1.492308 | high |
| GSM4153870 | 0.832877 | 1 | 8.777705 | 11.76323 | 10.45726 | 10.58805 | 12.08262 | 1.360012 | high |
| GSM4153871 | 2.835616 | 1 | 10.61731 | 9.173096 | 10.98375 | 10.57029 | 12.21043 | 0.578328 | low |
| GSM4153872 | 3.271233 | 1 | 9.73103 | 10.83066 | 10.59778 | 12.36683 | 12.58529 | 0.726375 | low |
| GSM4153873 | 1.032877 | 1 | 8.542187 | 11.11812 | 10.16722 | 8.760218 | 12.36683 | 1.130983 | high |
| GSM4153874 | 2.093151 | 1 | 9.484866 | 11.50557 | 11.20606 | 9.572622 | 12.44482 | 1.593314 | high |
| GSM4153875 | 2.871233 | 0 | 10.90014 | 10.28766 | 10.44034 | 12.24133 | 12.14214 | 0.543737 | low |
| GSM4153876 | 2.561644 | 0 | 9.790093 | 10.14309 | 10.97423 | 11.55354 | 12.72392 | 0.714588 | low |
| GSM4153877 | 2.583562 | 0 | 8.748728 | 12.44021 | 10.67645 | 10.52086 | 12.97992 | 1.539908 | high |
| GSM4153878 | 2.145205 | 1 | 8.145081 | 11.86449 | 9.535905 | 11.47978 | 11.3174 | 1.120017 | high |
| GSM4153879 | 2.517808 | 0 | 6.224367 | 8.769378 | 11.32452 | 5.307706 | 12.40261 | 1.663549 | high |
| GSM4153880 | 2.457534 | 1 | 9.621732 | 10.14595 | 11.26207 | 5.479423 | 12.4755 | 1.516009 | high |
| GSM4153881 | 2.567123 | 1 | 8.765972 | 11.71786 | 10.20734 | 8.597901 | 12.40301 | 1.353704 | high |
| GSM4153882 | 2.457534 | 0 | 7.96906 | 10.37553 | 11.14648 | 8.90451 | 13.09349 | 1.246912 | high |
| GSM4153883 | 1.857534 | 1 | 8.079136 | 11.6331 | 11.62478 | 11.14477 | 12.46565 | 2.068407 | high |
| GSM4153884 | 0.364384 | 1 | 9.529105 | 10.38306 | 12.11564 | 11.82043 | 12.63685 | 1.287571 | high |
| GSM4153885 | 1.6 | 1 | 10.23631 | 9.571966 | 11.43271 | 10.10563 | 12.84237 | 0.761832 | low |
| GSM4153886 | 1.761644 | 0 | 10.36585 | 9.767834 | 10.68641 | 11.85663 | 12.78531 | 0.499784 | low |
| GSM4153887 | 1.542466 | 0 | 10.56744 | 10.57318 | 11.22108 | 5.349273 | 12.36186 | 1.550034 | high |
| GSM4153888 | 2.216438 | 0 | 9.933542 | 10.03644 | 11.31533 | 9.818016 | 12.19097 | 1.027039 | low |
| GSM4153889 | 2.016438 | 0 | 7.552314 | 10.56838 | 11.39544 | 10.05618 | 12.235 | 1.679766 | high |
| GSM4153890 | 0.961644 | 1 | 9.628917 | 9.618645 | 10.72263 | 10.51856 | 12.57979 | 0.631952 | low |
| GSM4153891 | 1.805479 | 0 | 9.538973 | 10.62129 | 11.03901 | 10.02769 | 12.3435 | 1.097583 | high |
| GSM4153892 | 1.778082 | 0 | 8.809042 | 11.13184 | 10.42066 | 11.06272 | 12.54317 | 0.952814 | low |
| GSM4153893 | 2.742466 | 0 | 9.98376 | 11.11175 | 11.12083 | 10.80792 | 12.94442 | 1.022058 | low |
| GSM4153894 | 3.145205 | 0 | 9.894808 | 8.2659 | 11.5477 | 11.38689 | 12.62728 | 0.520346 | low |
| GSM4153895 | 3.060274 | 0 | 9.584348 | 11.07081 | 10.50609 | 10.22224 | 12.57676 | 0.935296 | low |
| GSM4153896 | 2.189041 | 1 | 9.916334 | 11.16413 | 10.5818 | 10.77563 | 13.04266 | 0.81915 | low |
| GSM4153897 | 2.049315 | 0 | 10.23762 | 11.74933 | 10.44321 | 9.753512 | 12.35196 | 1.125216 | high |
| GSM4153898 | 3.09589 | 0 | 8.777705 | 9.271316 | 10.79468 | 12.59836 | 12.01755 | 0.611717 | low |
| GSM4153899 | 3.073973 | 0 | 8.179059 | 10.03163 | 9.646668 | 11.24449 | 11.98826 | 0.587214 | low |
| GSM4153900 | 2.991781 | 0 | 9.800684 | 10.26625 | 9.68542 | 11.79327 | 12.46791 | 0.44274 | low |
| GSM4153901 | 1.827397 | 1 | 6.707211 | 8.802536 | 10.71887 | 5.55671 | 11.98394 | 1.299117 | high |
| GSM4153902 | 0.386301 | 1 | 9.612882 | 10.99596 | 11.00705 | 11.28068 | 12.66295 | 1.004198 | low |
| GSM4153903 | 2.024658 | 0 | 5.447461 | 11.76781 | 9.968067 | 10.24434 | 12.37047 | 1.690338 | high |
| GSM4153904 | 3.230137 | 0 | 9.37891 | 9.444007 | 11.33904 | 11.98502 | 12.12542 | 0.774709 | low |
| GSM4153905 | 2.347945 | 0 | 9.923559 | 10.79996 | 11.29349 | 12.32989 | 11.87507 | 1.097483 | high |
| GSM4153906 | 2.271233 | 1 | 9.528427 | 9.882298 | 11.94816 | 11.92579 | 12.29331 | 1.093215 | high |
| GSM4153907 | 1.687671 | 1 | 7.591366 | 11.63379 | 10.5426 | 11.91053 | 12.23894 | 1.365525 | high |
| GSM4153908 | 1.742466 | 0 | 10.31557 | 9.492087 | 9.313086 | 11.49081 | 12.67362 | 0.272861 | low |
| GSM4153909 | 1.649315 | 0 | 9.479765 | 10.93262 | 9.814343 | 10.90245 | 11.91419 | 0.732036 | low |
| GSM4153910 | 2.6 | 1 | 9.262864 | 11.50593 | 12.00345 | 10.67942 | 12.42121 | 2.09371 | high |
| GSM4153911 | 1.649315 | 1 | 8.57114 | 11.56516 | 10.34594 | 9.919283 | 12.22758 | 1.293487 | high |
| GSM4153912 | 3.224658 | 0 | 8.703612 | 10.33352 | 12.10996 | 9.300878 | 12.64017 | 1.779432 | high |
| GSM4153913 | 2.638356 | 0 | 8.816666 | 8.366416 | 10.43119 | 11.78577 | 12.37492 | 0.393388 | low |
| GSM4153914 | 2.386301 | 0 | 9.629581 | 9.823423 | 11.32994 | 10.21772 | 12.8531 | 0.84566 | low |
| GSM4153915 | 2.235616 | 0 | 5.536222 | 10.89716 | 9.538973 | 10.83833 | 12.71424 | 0.936186 | low |
| GSM4153916 | 2.150685 | 0 | 10.41836 | 12.14452 | 10.50444 | 11.45756 | 12.40459 | 1.078432 | high |
| GSM4153917 | 2.060274 | 0 | 10.01174 | 9.447384 | 10.67809 | 11.8898 | 12.31249 | 0.521109 | low |
| GSM4153918 | 1.841096 | 0 | 8.361408 | 11.24113 | 10.55626 | 10.68072 | 12.39343 | 1.185788 | high |
| GSM4153919 | 2.145205 | 1 | 10.27539 | 10.59377 | 9.754219 | 12.77781 | 11.86133 | 0.491331 | low |
| GSM4153920 | 2.906849 | 0 | 8.339213 | 11.62141 | 9.474254 | 9.726973 | 12.65046 | 0.873321 | low |
| GSM4153921 | 1.980822 | 1 | 6.812917 | 12.43262 | 10.33678 | 9.298389 | 12.66249 | 2.068272 | high |
| GSM4153922 | 2.789041 | 0 | 8.605294 | 12.14059 | 10.1391 | 11.14684 | 12.55697 | 1.175875 | high |
| GSM4153923 | 3.054795 | 0 | 10.45394 | 12.33151 | 11.40734 | 10.86617 | 12.52884 | 1.715975 | high |
| GSM4153924 | 3.153425 | 0 | 8.987727 | 10.7379 | 11.36622 | 11.72839 | 12.34506 | 1.20671 | high |
| GSM4153925 | 2.216438 | 1 | 7.931038 | 10.85356 | 10.52311 | 10.55366 | 12.79471 | 1.022046 | low |
| GSM4153926 | 2.580822 | 0 | 9.574642 | 9.755183 | 10.06457 | 10.54751 | 12.99224 | 0.459218 | low |
| GSM4153927 | 2.265753 | 0 | 7.625443 | 11.80016 | 10.38719 | 9.917343 | 10.54819 | 2.29354 | high |
| GSM4153928 | 1.986301 | 0 | 8.931381 | 11.25969 | 11.23235 | 8.10437 | 12.63974 | 1.766262 | high |
| GSM4153929 | 1.706849 | 1 | 8.483938 | 12.22331 | 11.33833 | 9.555581 | 12.57637 | 2.350473 | high |
| GSM4153930 | 1.421918 | 0 | 9.824441 | 10.54029 | 8.556688 | 11.14858 | 12.2726 | 0.32869 | low |
| GSM4153931 | 2.887671 | 0 | 5.687581 | 11.44938 | 8.737759 | 11.37325 | 12.76847 | 0.729174 | low |
| GSM4153932 | 0.479452 | 0 | 9.433291 | 9.667483 | 9.967755 | 11.37122 | 12.41475 | 0.458014 | low |
| GSM4153933 | 2.476712 | 0 | 11.1924 | 8.894006 | 10.40939 | 10.65232 | 12.33973 | 0.37117 | low |
| GSM4153934 | 2.249315 | 0 | 8.348372 | 9.883606 | 11.14954 | 12.14794 | 12.69007 | 0.824514 | low |
| GSM4153935 | 1.986301 | 0 | 9.121055 | 11.48529 | 10.15036 | 11.51144 | 12.43304 | 0.892497 | low |
| GSM4153936 | 1.978082 | 0 | 7.318751 | 12.30917 | 10.20894 | 10.704 | 11.86664 | 1.83068 | high |
| GSM4153937 | 1.487671 | 0 | 8.097212 | 10.73057 | 10.41934 | 8.042952 | 12.74816 | 1.168447 | high |
| GSM4153938 | 3.021918 | 0 | 7.888518 | 10.88203 | 10.81343 | 5.551717 | 12.29255 | 2.05748 | high |
| GSM4153939 | 2.956164 | 0 | 9.066235 | 9.723246 | 10.28267 | 12.37736 | 12.29869 | 0.523541 | low |
| GSM4153940 | 2.947945 | 0 | 7.614804 | 10.08934 | 10.02901 | 10.28832 | 13.13225 | 0.651213 | low |
| GSM4153941 | 1.682192 | 1 | 7.27574 | 10.81147 | 10.88832 | 9.644667 | 12.71098 | 1.424126 | high |
| GSM4153942 | 1.978082 | 0 | 5.630543 | 11.48971 | 10.38404 | 5.383762 | 12.76752 | 2.585814 | high |
| GSM4153943 | 2.876712 | 0 | 6.387276 | 10.35511 | 9.287547 | 10.15701 | 12.89957 | 0.648218 | low |
| GSM4153944 | 1.769863 | 1 | 7.328951 | 9.71285 | 11.62038 | 10.73415 | 12.15341 | 1.397477 | high |
| GSM4153945 | 2.09589 | 0 | 5.702256 | 10.82475 | 10.14244 | 5.810185 | 12.9591 | 1.737144 | high |
| GSM4153946 | 1.578082 | 1 | 5.96203 | 11.49499 | 11.39304 | 5.398088 | 12.68635 | 3.861285 | high |
| GSM4153947 | 2 | 0 | 5.519938 | 10.65756 | 11.06005 | 5.204512 | 12.26541 | 3.053407 | high |
| GSM4153948 | 1.772603 | 0 | 10.42837 | 8.082637 | 11.26438 | 12.91154 | 12.3532 | 0.373683 | low |
| GSM4153949 | 2.616438 | 1 | 10.10725 | 10.9021 | 11.23674 | 10.33386 | 12.5785 | 1.11571 | high |
| GSM4153950 | 2.328767 | 1 | 5.585838 | 12.07173 | 10.49617 | 11.6496 | 12.89271 | 1.797947 | high |
| GSM4153951 | 1.90137 | 0 | 8.986317 | 9.256173 | 10.29062 | 10.64312 | 12.60429 | 0.50462 | low |
| GSM4153952 | 0.980822 | 1 | 9.373873 | 7.413721 | 9.807441 | 10.72227 | 12.45675 | 0.225498 | low |
| GSM4153953 | 2.139726 | 1 | 5.7293 | 8.394255 | 11.21609 | 10.50542 | 11.70522 | 1.090739 | high |
| GSM4153954 | 2.09589 | 0 | 9.200141 | 11.35873 | 10.59075 | 11.06205 | 12.32326 | 1.091356 | high |
| GSM4153955 | 2.065753 | 0 | 9.126345 | 9.632951 | 11.00962 | 11.45756 | 12.73256 | 0.682512 | low |
| GSM4153956 | 0.336986 | 1 | 10.01331 | 10.11056 | 10.9498 | 10.71293 | 12.08635 | 0.838586 | low |
| GSM4153957 | 2.813699 | 1 | 9.970019 | 9.692323 | 12.42838 | 9.435075 | 12.31448 | 1.486997 | high |
| GSM4153958 | 2.690411 | 0 | 10.23762 | 11.72269 | 9.717141 | 11.25835 | 12.61673 | 0.675934 | low |
| GSM4153959 | 0.567123 | 0 | 5.64337 | 11.92471 | 10.0597 | 10.0336 | 13.07282 | 1.579674 | high |
| GSM4153960 | 1.076712 | 1 | 10.09908 | 9.792758 | 10.93561 | 11.56413 | 12.12318 | 0.685478 | low |
| GSM4153961 | 1.783562 | 0 | 10.03095 | 11.4846 | 10.56377 | 10.32179 | 12.45847 | 1.04184 | high |
| GSM4153962 | 1.569863 | 0 | 8.579681 | 9.633305 | 11.01061 | 10.98241 | 12.51647 | 0.803915 | low |
| GSM4153963 | 2.745205 | 0 | 7.654779 | 10.80129 | 10.68541 | 8.194701 | 12.65919 | 1.42721 | high |
| GSM4153964 | 2.183562 | 0 | 10.75928 | 11.8718 | 10.16428 | 11.66393 | 13.00941 | 0.707456 | low |
| GSM4153965 | 3.115068 | 0 | 11.92618 | 8.042563 | 10.72655 | 12.5086 | 12.28659 | 0.252013 | low |
| GSM4153966 | 2.717808 | 0 | 9.866595 | 10.70532 | 10.54453 | 10.52811 | 12.59159 | 0.791905 | low |
| GSM4153967 | 3.112329 | 0 | 10.12464 | 7.344477 | 10.90375 | 11.27218 | 12.30362 | 0.311851 | low |
| GSM4153968 | 2.534247 | 0 | 10.13347 | 12.18982 | 10.80094 | 11.10245 | 11.82837 | 1.503725 | high |
| GSM4153969 | 2.126027 | 1 | 6.015455 | 10.39726 | 9.800022 | 5.779969 | 10.00716 | 2.353139 | high |
| GSM4153970 | 2.29589 | 1 | 10.26258 | 10.75693 | 9.224107 | 11.03372 | 12.47297 | 0.426031 | low |
| GSM4153971 | 2.30411 | 0 | 7.310081 | 10.3149 | 11.09435 | 10.14967 | 12.51775 | 1.31924 | high |
| GSM4153972 | 2.819178 | 0 | 9.939722 | 10.28733 | 11.77106 | 9.834033 | 12.50901 | 1.257129 | high |
| GSM4153973 | 3.005479 | 0 | 10.60281 | 8.981061 | 11.1252 | 11.4046 | 12.51172 | 0.504299 | low |
| GSM4153974 | 2.50411 | 0 | 5.706338 | 11.94744 | 11.91234 | 5.485711 | 13.12173 | 5.191709 | high |
| GSM4153975 | 0.019178 | 0 | 10.23237 | 10.65201 | 11.12954 | 5.641832 | 12.83328 | 1.409887 | high |
| GSM4153976 | 1.969863 | 0 | 9.800022 | 10.93262 | 9.674412 | 12.29135 | 11.98864 | 0.572788 | low |
| GSM4153977 | 1.838356 | 0 | 7.193892 | 11.76104 | 10.91134 | 8.749441 | 13.19242 | 1.914015 | high |
| GSM4153978 | 2.652055 | 1 | 8.637318 | 10.80426 | 10.92329 | 9.669823 | 11.27866 | 1.616308 | high |
| GSM4153979 | 2.953425 | 0 | 8.963343 | 10.24466 | 11.51275 | 11.03802 | 12.84704 | 1.059484 | high |
| GSM4153980 | 2.860274 | 0 | 9.580064 | 9.692 | 11.44834 | 12.60342 | 12.50146 | 0.744651 | low |
| GSM4153981 | 2.736986 | 0 | 5.307508 | 11.12083 | 10.38008 | 11.23641 | 12.6826 | 1.436511 | high |
| GSM4153982 | 2.652055 | 0 | 9.308498 | 8.525434 | 11.50284 | 11.65131 | 12.72392 | 0.573203 | low |
| GSM4153983 | 0.134247 | 0 | 5.694107 | 9.550853 | 9.795136 | 11.29752 | 12.36313 | 0.69486 | low |
| GSM4153984 | 0.978082 | 1 | 8.262302 | 10.73612 | 10.02475 | 9.798089 | 12.76509 | 0.82179 | low |
| GSM4153985 | 1.983562 | 0 | 7.525038 | 10.45953 | 10.87272 | 12.0194 | 12.89271 | 0.950392 | low |
| GSM4153986 | 2.008219 | 0 | 6.064511 | 11.72269 | 11.79759 | 11.10646 | 12.81342 | 2.807659 | high |
| GSM4153987 | 2.841096 | 0 | 9.399972 | 8.387498 | 10.27077 | 10.43347 | 11.82911 | 0.43355 | low |
| GSM4153988 | 1.813699 | 1 | 6.837499 | 11.07247 | 11.14142 | 8.915558 | 12.22798 | 2.159288 | high |
| GSM4153989 | 2.791781 | 0 | 11.14043 | 10.31126 | 9.492792 | 10.92632 | 12.41057 | 0.377641 | low |
| GSM4153990 | 3.060274 | 0 | 11.39748 | 9.445022 | 10.15135 | 11.1021 | 12.30399 | 0.37112 | low |
| GSM4153991 | 2.323288 | 0 | 10.30767 | 9.582069 | 10.63748 | 10.81647 | 12.37816 | 0.557933 | low |
| GSM4153992 | 2.079452 | 0 | 8.844231 | 12.20275 | 10.44417 | 10.87772 | 12.67932 | 1.319375 | high |
| GSM4153993 | 2.819178 | 0 | 9.438205 | 10.35831 | 10.96003 | 11.43238 | 12.37492 | 0.866792 | low |
| GSM4153994 | 2.835616 | 0 | 8.904161 | 11.05899 | 8.749837 | 10.81278 | 11.90588 | 0.529025 | low |
| GSM4153995 | 2.410959 | 0 | 9.885279 | 9.819379 | 11.90982 | 10.9097 | 13.30337 | 0.891178 | low |
| GSM4153996 | 2.115068 | 0 | 10.17702 | 9.97196 | 11.07633 | 9.973317 | 12.4955 | 0.813642 | low |
| GSM4153997 | 1.90137 | 0 | 9.393084 | 10.58044 | 10.21412 | 11.33128 | 12.46791 | 0.673569 | low |
| GSM4153998 | 1.378082 | 1 | 9.284056 | 12.00423 | 9.86596 | 11.78894 | 12.25275 | 0.920211 | low |
| GSM4153999 | 1.789041 | 0 | 7.400605 | 10.44715 | 11.5744 | 8.25021 | 12.8199 | 1.857536 | high |
| GSM4154000 | 0.786301 | 1 | 9.589095 | 11.86959 | 11.5779 | 10.78653 | 12.31613 | 1.894398 | high |
| GSM4154001 | 2.942466 | 0 | 8.704771 | 9.749784 | 9.877414 | 10.31853 | 12.73652 | 0.513576 | low |
| GSM4154002 | 2.479452 | 0 | 8.475901 | 10.47164 | 10.29029 | 10.48514 | 12.65 | 0.792193 | low |
| GSM4154003 | 0.958904 | 1 | 5.401735 | 11.15907 | 10.47297 | 9.469864 | 12.92313 | 1.66719 | high |
| GSM4154004 | 1.832877 | 1 | 9.048127 | 11.63066 | 11.36316 | 9.499927 | 12.57637 | 1.839877 | high |
| GSM4154005 | 1.627397 | 1 | 6.844767 | 11.36252 | 11.45823 | 9.735597 | 12.70433 | 2.26661 | high |
| GSM4154006 | 2.657534 | 0 | 9.57364 | 11.24783 | 9.124168 | 12.74183 | 12.44237 | 0.449767 | low |
| GSM4154007 | 1.021918 | 1 | 9.551843 | 8.770097 | 9.936155 | 11.01527 | 12.45547 | 0.344793 | low |
| GSM4154008 | 2.252055 | 0 | 9.614253 | 11.1924 | 10.8675 | 12.0128 | 12.7316 | 0.927218 | low |
| GSM4154009 | 2.172603 | 0 | 10.14375 | 10.05145 | 8.960449 | 11.094 | 12.90429 | 0.282481 | low |
| GSM4154010 | 1.627397 | 1 | 9.656719 | 10.27406 | 9.542055 | 10.89716 | 12.43685 | 0.465133 | low |
| GSM4154011 | 0.747945 | 1 | 9.310922 | 10.12863 | 10.4877 | 10.17118 | 12.13592 | 0.792769 | low |
| GSM4154012 | 1.715068 | 0 | 7.857345 | 10.5569 | 10.889 | 5.258097 | 12.53373 | 1.883258 | high |
| GSM4154013 | 2.764384 | 0 | 9.939383 | 10.4783 | 11.75176 | 11.75002 | 12.31573 | 1.157086 | high |
| GSM4154014 | 2.758904 | 0 | 8.909956 | 9.822795 | 10.72263 | 11.64997 | 12.33726 | 0.704749 | low |
| GSM4154015 | 2.821918 | 0 | 10.40048 | 11.07048 | 11.23407 | 9.676166 | 12.33809 | 1.260166 | high |
| GSM4154016 | 2.734247 | 0 | 9.05397 | 10.76122 | 9.889237 | 12.43771 | 12.32326 | 0.605677 | low |
| GSM4154017 | 0.926027 | 1 | 8.24495 | 11.95073 | 10.8267 | 8.454225 | 12.46999 | 2.030045 | high |
| GSM4154018 | 2.073973 | 0 | 8.801008 | 10.67741 | 10.655 | 9.577057 | 12.08451 | 1.154174 | high |
| GSM4154019 | 1.769863 | 0 | 7.300458 | 10.37392 | 11.11608 | 5.65668 | 11.56284 | 2.500595 | high |
| GSM4154020 | 1.917808 | 0 | 7.300057 | 11.52074 | 10.37104 | 10.85629 | 11.99042 | 1.479431 | high |
| GSM4154021 | 1.747945 | 0 | 5.534809 | 11.18171 | 10.16532 | 11.67635 | 12.42838 | 1.31304 | high |
| GSM4154022 | 2.824658 | 0 | 9.969389 | 10.30081 | 10.80824 | 8.617594 | 12.35115 | 0.964718 | low |
| GSM4154023 | 2.690411 | 0 | 7.830221 | 9.539311 | 10.27769 | 10.45887 | 12.91104 | 0.611323 | low |
| GSM4154024 | 2.764384 | 0 | 8.804795 | 10.86294 | 11.01994 | 10.40017 | 12.21274 | 1.288396 | high |
| GSM4154025 | 1.484932 | 1 | 8.661064 | 9.601349 | 11.00337 | 11.5577 | 12.42929 | 0.758495 | low |
| GSM4154026 | 1.350685 | 0 | 10.13347 | 11.77428 | 10.28503 | 11.39748 | 12.12811 | 0.970375 | low |
| GSM4154027 | 2.690411 | 0 | 9.977878 | 9.370752 | 10.15001 | 10.86092 | 12.78981 | 0.405771 | low |
| GSM4154028 | 2.443836 | 0 | 10.09158 | 10.21184 | 8.815145 | 12.50324 | 12.53823 | 0.267013 | low |
| GSM4154029 | 2.572603 | 0 | 5.349441 | 8.811299 | 10.91663 | 11.61031 | 12.79959 | 0.826585 | low |
| GSM4154030 | 2.293151 | 0 | 9.245771 | 12.3877 | 10.5631 | 10.2606 | 12.5826 | 1.502348 | high |
| GSM4154031 | 2.460274 | 0 | 11.15223 | 11.58098 | 10.63349 | 11.09602 | 12.20655 | 0.932753 | low |
| GSM4154032 | 2.271233 | 0 | 5.861314 | 8.606441 | 10.22452 | 10.68479 | 12.14017 | 0.674049 | low |
| GSM4154033 | 2.254795 | 0 | 9.999179 | 9.362721 | 10.66862 | 11.09705 | 12.07098 | 0.572779 | low |
| GSM4154034 | 2.268493 | 0 | 8.822107 | 10.40974 | 10.0472 | 10.76948 | 12.53599 | 0.667348 | low |
| GSM4154035 | 2.232877 | 0 | 8.026185 | 10.05746 | 12.15031 | 9.856637 | 12.39183 | 1.825048 | high |
| GSM4154036 | 0.452055 | 0 | 8.136057 | 9.317346 | 10.59811 | 10.80857 | 12.4381 | 0.67114 | low |
| GSM4154037 | 2.076712 | 0 | 6.93554 | 11.54288 | 10.05212 | 10.43902 | 13.03768 | 1.139085 | high |
| GSM4154038 | 2.161644 | 0 | 5.222979 | 11.08574 | 10.52645 | 12.11302 | 11.97339 | 1.6395 | high |
| GSM4154039 | 2.131507 | 0 | 10.99066 | 9.876416 | 9.764452 | 10.98804 | 12.82439 | 0.344516 | low |
| GSM4154040 | 2.180822 | 0 | 10.41769 | 11.477 | 9.949034 | 10.84793 | 12.42329 | 0.72928 | low |
| GSM4154041 | 1.742466 | 0 | 10.56443 | 11.30653 | 10.88173 | 11.2468 | 12.36481 | 0.983779 | low |
| GSM4154042 | 1.950685 | 0 | 8.967431 | 10.67483 | 11.54288 | 11.4812 | 12.29094 | 1.323232 | high |
| GSM4154043 | 1.808219 | 0 | 9.746116 | 10.43602 | 10.98271 | 10.32212 | 12.95375 | 0.842196 | low |
| GSM4154044 | 1.578082 | 0 | 6.053144 | 10.49393 | 10.64181 | 9.792092 | 12.5556 | 1.399295 | high |
| GSM4154045 | 0.687671 | 1 | 6.215828 | 8.06793 | 10.56242 | 9.963173 | 12.39103 | 0.635623 | low |
| GSM4154046 | 2.263014 | 0 | 10.13847 | 7.524656 | 9.610174 | 12.81683 | 12.02309 | 0.175074 | low |
| GSM4154047 | 2.210959 | 0 | 8.784188 | 10.51292 | 10.80361 | 10.2847 | 12.60472 | 0.983908 | low |
| GSM4154048 | 0.054795 | 0 | 10.50677 | 11.84876 | 11.71338 | 10.90735 | 12.5583 | 1.653921 | high |
| GSM4154049 | 2.232877 | 0 | 6.308891 | 11.64718 | 10.29191 | 12.55654 | 12.79325 | 1.231331 | high |
| GSM4154050 | 2.534247 | 0 | 10.72295 | 9.25476 | 10.56595 | 11.63413 | 13.1937 | 0.361274 | low |
| GSM4154051 | 2.290411 | 0 | 7.956349 | 10.86884 | 10.8463 | 10.6936 | 12.28659 | 1.289948 | high |
| GSM4154052 | 2.479452 | 0 | 8.093528 | 10.3635 | 10.03837 | 10.47265 | 12.61352 | 0.731497 | low |
| GSM4154053 | 2.339726 | 0 | 9.027108 | 10.76289 | 11.42477 | 5.130285 | 12.93359 | 1.999336 | high |
| GSM4154054 | 1.435616 | 1 | 8.703264 | 9.643659 | 11.1614 | 11.22684 | 12.44771 | 0.838938 | low |
| GSM4154055 | 2.556164 | 0 | 5.468674 | 8.577278 | 9.572622 | 12.23731 | 12.47467 | 0.432158 | low |
| GSM4154056 | 1.854795 | 0 | 8.590828 | 10.76584 | 11.59724 | 8.573338 | 12.33683 | 1.892836 | high |
| GSM4154057 | 2.564384 | 0 | 9.154748 | 10.06822 | 9.928598 | 11.08233 | 12.79374 | 0.502194 | low |
| GSM4154058 | 2.260274 | 0 | 10.042 | 9.393776 | 10.51357 | 10.68243 | 12.4533 | 0.51582 | low |
| GSM4154059 | 0.676712 | 1 | 9.385881 | 9.366195 | 10.47231 | 10.78524 | 12.71766 | 0.514872 | low |
| GSM4154060 | 1.767123 | 0 | 9.724561 | 11.85095 | 11.38722 | 10.67676 | 12.47856 | 1.664457 | high |
| GSM4154061 | 1.767123 | 0 | 5.276641 | 12.06093 | 9.28374 | 9.996224 | 12.44732 | 1.425888 | high |
| GSM4154062 | 0.493151 | 1 | 7.946399 | 12.2116 | 10.23106 | 10.81309 | 12.23617 | 1.507874 | high |
| GSM4154063 | 2.054795 | 0 | 8.142273 | 12.34589 | 11.30785 | 9.650771 | 11.94781 | 2.857083 | high |
| GSM4154064 | 2.312329 | 0 | 9.922249 | 12.00423 | 9.080694 | 10.9987 | 12.15567 | 0.662924 | low |
| GSM4154065 | 1.693151 | 0 | 8.286638 | 10.80758 | 10.33616 | 7.833802 | 12.42838 | 1.227316 | high |
| GSM4154066 | 0.983562 | 1 | 10.2437 | 10.64115 | 10.17085 | 10.28961 | 12.46521 | 0.660394 | low |
| GSM4154067 | 1.10411 | 1 | 5.560835 | 10.35635 | 11.33499 | 11.13646 | 12.58663 | 1.691578 | high |
| GSM4154068 | 2.635616 | 0 | 5.503888 | 12.49979 | 8.853077 | 10.11285 | 13.09185 | 1.139631 | high |
| GSM4154069 | 2.123288 | 0 | 7.895227 | 11.54735 | 10.43218 | 5.648108 | 12.76032 | 1.930284 | high |
| GSM4154070 | 1.564384 | 0 | 10.65098 | 8.58545 | 10.27832 | 11.23506 | 12.5391 | 0.312267 | low |
| GSM4154071 | 1.564384 | 1 | 10.5426 | 11.91272 | 10.67222 | 12.15994 | 12.06763 | 1.067051 | high |
| GSM4154072 | 0.00274 | 0 | 10.16163 | 7.594096 | 10.24559 | 11.19208 | 12.31407 | 0.25478 | low |
| GSM4154073 | 2.49863 | 0 | 8.63186 | 10.36938 | 10.19009 | 12.10844 | 12.24366 | 0.676444 | low |
| GSM4154074 | 2.169863 | 1 | 8.402992 | 11.98179 | 11.31128 | 8.940183 | 12.54364 | 2.321553 | high |
| GSM4154075 | 1.684932 | 1 | 9.516183 | 11.52245 | 10.29029 | 9.584348 | 12.88377 | 0.984409 | low |
| GSM4154076 | 2.131507 | 0 | 7.843302 | 10.27238 | 10.47265 | 10.28232 | 12.53599 | 0.915103 | low |
| GSM4154077 | 2.191781 | 0 | 9.314833 | 11.56383 | 11.25058 | 10.38437 | 12.46434 | 1.564888 | high |
| GSM4154078 | 2.569863 | 0 | 8.889534 | 9.7705 | 10.3989 | 10.86392 | 12.92783 | 0.574916 | low |
| GSM4154079 | 2.580822 | 0 | 9.213494 | 11.20065 | 11.30817 | 10.98742 | 12.71566 | 1.304159 | high |
| GSM4154080 | 0.928767 | 1 | 10.40872 | 10.02082 | 11.40188 | 5.457697 | 12.86942 | 1.282304 | high |
| GSM4154081 | 1.517808 | 0 | 5.882541 | 11.14343 | 11.57276 | 11.43986 | 12.71766 | 2.16351 | high |
| GSM4154082 | 1.479452 | 0 | 7.963069 | 9.941347 | 11.08336 | 11.72482 | 12.71424 | 0.889353 | low |
| GSM4154083 | 0.950685 | 1 | 8.061601 | 11.3428 | 11.20976 | 8.085916 | 12.58529 | 2.047293 | high |
| GSM4154084 | 2.186301 | 0 | 10.1951 | 10.35048 | 10.74882 | 5.356475 | 13.34081 | 1.012596 | low |
| GSM4154085 | 2.358904 | 0 | 10.38271 | 10.71595 | 10.09807 | 10.44131 | 12.51775 | 0.627231 | low |
| GSM4154086 | 1.808219 | 0 | 9.168576 | 10.88535 | 10.5572 | 8.975572 | 12.86574 | 1.006722 | low |
| GSM4154087 | 2.109589 | 0 | 8.767112 | 8.838522 | 9.935185 | 12.28295 | 12.60382 | 0.338227 | low |
| GSM4154088 | 1.452055 | 1 | 9.750447 | 11.38183 | 11.52178 | 8.555533 | 12.67174 | 1.768962 | high |
| GSM4154089 | 0.591781 | 1 | 10.22189 | 11.17694 | 11.23541 | 11.64894 | 12.36927 | 1.10817 | high |
| GSM4154090 | 1.947945 | 0 | 5.594176 | 11.24713 | 9.503705 | 12.23458 | 12.28774 | 0.982362 | low |
| GSM4154091 | 2.342466 | 0 | 8.155303 | 11.14311 | 10.47957 | 10.4197 | 12.98095 | 1.036242 | high |
| GSM4154092 | 2.235616 | 0 | 10.22093 | 8.611007 | 11.16918 | 11.31226 | 12.52705 | 0.485047 | low |
| GSM4154093 | 2.249315 | 1 | 5.629321 | 9.043124 | 11.89128 | 7.283606 | 12.27339 | 2.143908 | high |
| GSM4154094 | 2.268493 | 0 | 8.616415 | 10.52247 | 11.25197 | 9.801996 | 12.5378 | 1.294507 | high |
| GSM4154095 | 2.147945 | 0 | 8.250623 | 12.08863 | 10.50741 | 9.850287 | 12.94799 | 1.471841 | high |
| GSM4154096 | 2.032877 | 0 | 5.703372 | 11.52146 | 10.92527 | 5.541658 | 12.96233 | 3.078674 | high |
| GSM4154097 | 2 | 0 | 8.992106 | 11.18002 | 9.344161 | 11.05899 | 12.70715 | 0.577071 | low |
| GSM4154098 | 1.580822 | 0 | 5.91944 | 11.01494 | 10.48673 | 10.80395 | 10.68445 | 2.120134 | high |
| GSM4154099 | 0.29863 | 0 | 9.446385 | 10.31756 | 11.47562 | 10.94588 | 12.79471 | 1.018477 | low |
| GSM4154100 | 0.90411 | 0 | 7.207834 | 11.14922 | 9.932931 | 10.76979 | 12.58931 | 0.984816 | low |
| GSM4154101 | 1.813699 | 0 | 10.65944 | 9.561685 | 9.664412 | 5.272622 | 12.65369 | 0.546813 | low |
| GSM4154102 | 1.978082 | 0 | 8.336047 | 9.846995 | 10.48244 | 10.5901 | 12.66338 | 0.712923 | low |
| GSM4154103 | 0.328767 | 0 | 8.294137 | 10.05251 | 11.93368 | 11.16241 | 12.43346 | 1.410261 | high |
| GSM4154104 | 2.030137 | 0 | 7.750721 | 11.48083 | 11.76323 | 9.242952 | 12.66425 | 2.495866 | high |
| GSM4154105 | 1.756164 | 0 | 9.422715 | 11.43404 | 11.70096 | 11.37872 | 12.72303 | 1.55132 | high |
| GSM4154106 | 2.112329 | 0 | 9.798718 | 11.47088 | 11.96543 | 11.38148 | 12.71424 | 1.670837 | high |
| GSM4154107 | 1.594521 | 0 | 8.869572 | 11.84264 | 11.26305 | 5.300379 | 12.58843 | 2.822224 | high |
| GSM4154108 | 1.99726 | 0 | 10.63748 | 10.59546 | 7.615992 | 5.481367 | 11.98356 | 0.357631 | low |
| GSM4154109 | 1.750685 | 0 | 7.594856 | 10.74746 | 11.26074 | 11.00536 | 12.84498 | 1.344789 | high |
| GSM4154110 | 2 | 0 | 5.352755 | 10.1623 | 9.629581 | 10.70431 | 12.50367 | 0.840444 | low |
| GSM4154111 | 2.123288 | 0 | 9.270639 | 10.47957 | 11.79291 | 10.14833 | 12.97941 | 1.298331 | high |
| GSM4154112 | 0.758904 | 1 | 6.407906 | 11.52937 | 10.57029 | 10.87208 | 12.96233 | 1.483388 | high |
| GSM4154113 | 2.323288 | 0 | 10.68009 | 9.855651 | 9.801996 | 11.90947 | 12.33516 | 0.369771 | low |
| GSM4154114 | 2.109589 | 0 | 8.756035 | 9.612882 | 12.19682 | 10.19541 | 12.34229 | 1.438709 | high |
| GSM4154115 | 0.476712 | 0 | 8.592426 | 11.61238 | 10.19173 | 10.37362 | 12.10439 | 1.206939 | high |
| GSM4154116 | 1.726027 | 1 | 8.844633 | 11.06238 | 12.32492 | 10.17702 | 12.42754 | 2.314819 | high |
| GSM4154117 | 2.027397 | 0 | 9.346853 | 10.01526 | 11.79505 | 10.10918 | 12.2742 | 1.296485 | high |
| GSM4154118 | 1.687671 | 0 | 7.221458 | 10.52843 | 11.04274 | 11.49534 | 11.76323 | 1.447079 | high |
| GSM4154119 | 1.958904 | 0 | 8.216671 | 10.30705 | 11.24919 | 11.88768 | 11.736 | 1.250023 | high |
| GSM4154120 | 1.526027 | 0 | 11.23906 | 9.315189 | 8.757154 | 10.23337 | 12.77045 | 0.197661 | low |
| GSM4154121 | 1.380822 | 1 | 8.519971 | 10.30215 | 11.5042 | 8.805563 | 12.92147 | 1.377349 | high |
| GSM4154122 | 1.978082 | 0 | 9.808096 | 10.90014 | 11.50524 | 10.16657 | 12.2206 | 1.425101 | high |
| GSM4154123 | 1.849315 | 0 | 8.898495 | 11.77248 | 11.83053 | 9.775187 | 12.46107 | 2.391102 | high |
| GSM4154124 | 1.89589 | 0 | 8.660268 | 9.867575 | 11.03105 | 11.59202 | 12.26665 | 0.859945 | low |
| GSM4154125 | 1.767123 | 0 | 8.412091 | 10.7859 | 11.78502 | 11.41037 | 12.13904 | 1.701262 | high |
| GSM4154126 | 1.712329 | 0 | 11.08782 | 9.149742 | 11.01234 | 8.937618 | 12.26424 | 0.6255 | low |
| GSM4154127 | 2.271233 | 0 | 9.834033 | 10.8573 | 9.726973 | 5.361353 | 12.84755 | 0.894134 | low |
| GSM4154128 | 2.038356 | 0 | 9.026377 | 11.74012 | 11.61384 | 9.927214 | 12.04256 | 2.285321 | high |
| GSM4154129 | 1.065753 | 1 | 9.692639 | 8.719949 | 10.34625 | 11.96215 | 11.765 | 0.420513 | low |
| GSM4154130 | 0.561644 | 0 | 9.023775 | 10.16958 | 11.14545 | 9.900067 | 12.11906 | 1.13568 | high |
| GSM4154131 | 1.841096 | 0 | 10.25699 | 10.50542 | 11.40734 | 11.68097 | 12.14994 | 1.006003 | low |
| GSM4154132 | 1.589041 | 1 | 5.554803 | 11.95183 | 11.35504 | 7.814262 | 12.57364 | 3.801411 | high |
| GSM4154133 | 0.794521 | 1 | 8.363324 | 11.98644 | 10.75147 | 10.26092 | 12.39639 | 1.685139 | high |
| GSM4154134 | 1.424658 | 1 | 5.591646 | 11.27965 | 11.22041 | 7.352086 | 12.28096 | 3.217061 | high |
| GSM4154135 | 2.09589 | 0 | 11.09602 | 11.73739 | 10.51099 | 11.9559 | 12.06945 | 0.891328 | low |
| GSM4154136 | 0.643836 | 1 | 5.868035 | 12.19998 | 10.54485 | 10.06361 | 12.36396 | 2.374053 | high |
| GSM4154137 | 2.167123 | 0 | 11.5204 | 9.449386 | 10.69262 | 10.839 | 12.41817 | 0.459917 | low |
| GSM4154138 | 0.923288 | 0 | 10.13155 | 10.48313 | 11.18433 | 9.15614 | 11.75829 | 1.263683 | high |
| GSM4154139 | 1.671233 | 0 | 8.683824 | 11.39679 | 11.37457 | 12.78079 | 12.04183 | 1.4993 | high |
| GSM4154140 | 1.849315 | 0 | 11.03136 | 9.211018 | 10.88633 | 11.90222 | 11.86773 | 0.505169 | low |
| GSM4154141 | 1.906849 | 0 | 9.800022 | 9.919894 | 10.30705 | 12.52175 | 11.36383 | 0.611395 | low |
| GSM4154142 | 1.364384 | 0 | 7.962692 | 11.89312 | 10.44648 | 10.90177 | 12.4381 | 1.419984 | high |
| GSM4154143 | 2.09589 | 0 | 6.006198 | 10.63616 | 10.53038 | 7.547523 | 12.9724 | 1.577783 | high |
| GSM4154144 | 2.087671 | 0 | 5.317605 | 11.49568 | 10.4767 | 5.532198 | 12.54406 | 2.907635 | high |
| GSM4154145 | 1.791781 | 0 | 5.476176 | 11.17933 | 10.34301 | 11.3289 | 12.12134 | 1.57048 | high |
| GSM4154146 | 1.857534 | 0 | 7.986435 | 11.6045 | 11.10115 | 9.938394 | 12.93417 | 1.681075 | high |
| GSM4154147 | 1.624658 | 0 | 7.448027 | 11.68338 | 11.20065 | 10.39302 | 11.93977 | 2.287111 | high |
| GSM4154148 | 0.049315 | 0 | 7.738411 | 10.08634 | 11.17326 | 11.11276 | 11.98213 | 1.229214 | high |
| GSM4154149 | 1.712329 | 0 | 8.847274 | 11.69816 | 11.49534 | 9.954039 | 13.03982 | 1.777395 | high |
| GSM4154150 | 1.80274 | 0 | 5.45008 | 10.66503 | 11.06977 | 7.777154 | 12.9917 | 2.105971 | high |
| GSM4154151 | 1.30137 | 1 | 9.359553 | 11.65964 | 10.83973 | 9.520228 | 13.02229 | 1.294639 | high |
| GSM4154152 | 1.767123 | 0 | 8.551697 | 12.03319 | 10.43022 | 9.542055 | 12.04885 | 1.670194 | high |
| GSM4154153 | 1.747945 | 0 | 8.193834 | 11.40803 | 12.10957 | 9.184895 | 12.27858 | 2.90196 | high |
| GSM4154154 | 1.767123 | 0 | 8.460581 | 8.486286 | 11.26207 | 5.480241 | 12.19248 | 1.126255 | high |
| GSM4154155 | 1.756164 | 0 | 8.9682 | 10.69944 | 10.5426 | 10.02835 | 12.88955 | 0.877317 | low |
| GSM4154156 | 1.753425 | 0 | 9.379951 | 9.67648 | 10.45331 | 10.19702 | 12.4533 | 0.627757 | low |
| GSM4154157 | 1.561644 | 0 | 8.408543 | 11.36589 | 10.75147 | 12.61957 | 11.29553 | 1.4049 | high |
